# A Mathematical Model of Breast Tumor Progression Based on Immune Infiltration

**DOI:** 10.3390/jpm11101031

**Published:** 2021-10-15

**Authors:** Navid Mohammad Mirzaei, Sumeyye Su, Dilruba Sofia, Maura Hegarty, Mohamed H. Abdel-Rahman, Alireza Asadpoure, Colleen M. Cebulla, Young Hwan Chang, Wenrui Hao, Pamela R. Jackson, Adrian V. Lee, Daniel G. Stover, Zuzana Tatarova, Ioannis K. Zervantonakis, Leili Shahriyari

**Affiliations:** 1 Department of Mathematics and Statistics, University of Massachusetts Amherst, Amherst, MA 01003, USA; mirzaei@math.umass.edu (N.M.M.); sumeyyesu@umass.edu (S.S.); dsofia@umass.edu (D.S.); mshegarty@umass.edu (M.H.); 2 Department of Ophthalmology, Ohio State University Comprehensive Cancer Center, Columbus, OH 43210, USA; mohamed.abdel-rahman@osumc.edu (M.H.A.-R.); colleen.cebulla@osumc.edu (C.M.C.); Daniel.Stover@osumc.edu (D.G.S.); 3 Department of Civil and Environmental Engineering, University of Massachusetts, Dartmouth, MA 02747, USA; aasadpoure@umassd.edu; 4 Department of Biomedical Engineering and OHSU Center for Spatial Systems Biomedicine (OCSSB), Oregon Health and Science University, Portland, OR 97239, USA; chanyo@ohsu.edu (Y.H.C.); tatarova@ohsu.edu (Z.T.); 5 Department of Mathematics, The Pennsylvania State University, University Park, PA 16802, USA; wxh64@psu.edu; 6 Mathematical NeuroOncology Lab, Precision Neurotherapeutics Innovation Program, Mayo Clinic Arizona, Phoenix, AZ 85054, USA; Jackson.Pamela@mayo.edu; 7 Department of Pharmacology and Chemical Biology, University of Pittsburgh, Pittsburgh, PA 15219, USA; leeav@upmc.edu; 8 Department of Bioengineering, UPMC Hillman Cancer Center, University of Pittsburgh, Pittsburgh, PA 15219, USA; ioz1@pitt.edu

**Keywords:** breast cancer, immune infiltration, ordinary differential equations, data driven mathematical model, sensitivity analysis, adipocytes, estrogen, HMGB1, cytokines, T-cells, macrophages, IFN-γ

## Abstract

Breast cancer is the most prominent type of cancer among women. Understanding the microenvironment of breast cancer and the interactions between cells and cytokines will lead to better treatment approaches for patients. In this study, we developed a data-driven mathematical model to investigate the dynamics of key cells and cytokines involved in breast cancer development. We used gene expression profiles of tumors to estimate the relative abundance of each immune cell and group patients based on their immune patterns. Dynamical results show the complex interplay between cells and molecules, and sensitivity analysis emphasizes the direct effects of macrophages and adipocytes on cancer cell growth. In addition, we observed the dual effect of IFN-γ on cancer proliferation, either through direct inhibition of cancer cells or by increasing the cytotoxicity of CD8+ T-cells.

## 1. Introduction

Breast cancer is one of the most common cancers in women, and it is estimated that about 43,600 women in the United States will die because of breast cancer in 2021 [1]. There are different subtypes of breast cancer based on molecular-level analysis of gene expression patterns, such as luminal A (LumA), luminal B (LumB), and triple-negative/basal-like [2]. LumA is the most frequently seen subtype of breast cancer with the lowest mortality rate among other subtypes [3], and triple-negative breast cancer (TNBC) is the most aggressive subtype with a poor overall clinical outcome [4]. Several treatment options are available for breast cancer, including surgery (lumpectomy and mastectomy), radiotherapy, chemotherapy, hormone therapy, and other novel therapies that are determined by clinicopathological aspects of each patient [5]. Although targeted therapies have proven effective for some types of breast cancer [6], chemotherapy remains the standard form of treatment for subtypes of TNBC [7], and the development of resistance to chemotherapy is the main challenge for TNBCs [8,9].

It has been shown in many studies that the tumor’s microenvironment has a significant role in breast cancer development, progression, and also response to therapies [10,11,12,13]. Immune cells interact with tumor cells directly or indirectly via chemokine and cytokine signaling in the tumor microenvironment, and they are the major players in the behavior of the tumor and the efficiency of the treatments [14]. When cancer cells die, they release a protein called high mobility group box-1 (HMGB-1) that causes dendritic cells to become activated [15]. Antigens presented by dendritic cells cause T-cells to become activated and kill cancer directly [16,17,18]. IFN-γ is produced by helper T-cells, cytotoxic T-cells [19,20,21], and dendritic cells [19] to inhibit tumor growth [21]. On the other hand, certain immune cells can help cancer progress by either promoting it or having a dual impact. For example, regulatory T-cells control the development and activity of helper and cytotoxic T-cells, hence reducing the immune response and indirectly encouraging malignancy [22,23].

The relationship between clinical outcome and immune cells in breast tumor has been found in many studies. Breast cancer is distinguished by a significant population of tumor associated macrophages [24], and it has been shown that higher macrophage density is related to a poor outcome [25]. Moreover, the presence of CD8+ T-cells has been linked to considerable reductions in the relative risks of death from different subtypes of breast cancer [26]; and in ER-negative cancers, CD4+ and CD8+ T lymphocytes are more closely related to better outcomes than in ER-positive tumors [27]. In addition, it has been found that advanced stage breast tumors have more T-reg cells and a lower ratio of T-helper/T-reg cells [28]. All this evidence suggests that the relative numbers of distinct immune cells, and their interaction network, play key roles in the initiation and progression of breast cancer. Thus, to effectively simulate cancer progression, it is important to divide patients into similar cohorts based on their tumor-infiltrating immune cells and investigate the tumor progression of each group independently. However, adding all immune cells to the model increases the complexity and uncertainty of it. We therefore only considered the above-mentioned key immune cells (macrophages, CD4+ T-cells, T-reg cells, cytotoxic cells, and dendritic cells, which have a huge role in activating T-cells) in the progression of breast cancer.

Many mathematical models have been developed to study the relationships among tumors’ initiation, dynamics, and therapeutic responses to discover the best therapeutic combinations and overcome drug resistance in diverse cancer types [29,30,31,32,33,34,35,36,37], including breast cancer [38,39,40,41,42]. Some of the mathematical models for breast cancer investigate the relationships among immune cells, tumor cells, and some treatments [36,43,44]. For example, the effects of trastuzumab on HER2 overexpressing breast cancer in a mouse model system have been studied by integrating mathematical and experimental models [43]. In addition, a mathematical model has been developed to investigate the interactions between the MCF-7 breast cancer cell line and some immune cells. These models [43,45] include only a few immune components, such as NK cells and CD8+ T-cells, similarly to the mathematical model developed for the treatment of the murine 4T1 TNBC cell line with a high-dose chemotherapy drug [36].

**Figure 1:**
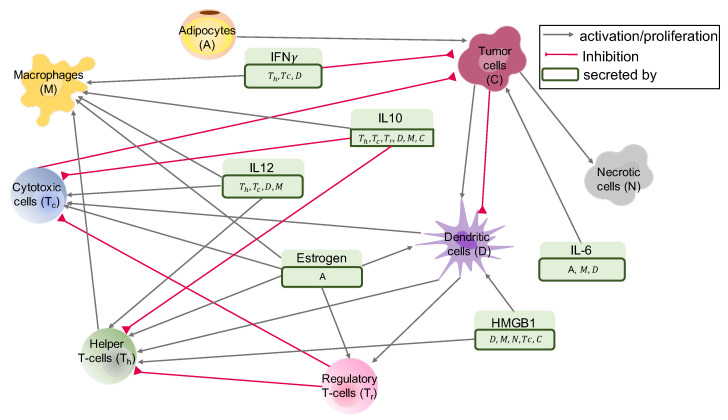
**Interaction network.** The main interaction network of cells and molecules in breast tumors, modeled in this paper. Variables of the model with their descriptions are given in Table 1.

Some of the outstanding challenges in the mathematical modeling of cancers are the existence of many unknown parameters and the limited number of datasets. For this reason, it is a routine practice to assume some values for parameters (see, e.g., [46,47,48]), or use estimated parameters from other diseases or models in the mathematical modeling of cancer. For example, the parameter values obtained for sarcoidosis were used to estimate the parameters of a mathematical model for pancreatic cancer, and the values estimated for pancreatic cancer in the mathematical modeling of breast cancer [23,49,50]. In addition, in a mathematical model of breast cancer [51], the values of some of parameters were chosen in line with a mathematical model of tumor invasion not validated for breast cancer [52]. Hence, the available mathematical models for breast cancer only consider a small subset of immune cells, and they assume all breast tumors behave similarly as they use the same parameter values for all tumors. However, since the evolution of a breast tumor depends on its specific immune profile, it is better to first find such tumors’ immune variations and understand the mechanism of growth in the absence of treatment for each of these immune variations. For this reason, we present a mathematical model for the interaction network given in Figure 1. We used a system of ordinary differential equations (ODEs) to investigate the differences in tumor progression of patients with different immune profiles. We clustered breast tumors based on their estimated immune cell frequencies using their gene expression data. We then estimated the parameters of the mathematical model for each tumor group separately. We found parameter values by reviewing the available literature and estimating the rest using what data were available. Importantly, we then performed global sensitivity analysis on the non-dimensionalized system to find the most sensitive parameters and investigate their impacts. Lastly, we analyzed the dynamics of the tumors in each group and compared them with patients’ clinical information to explore the connections between the tumor microenvironment and the progression of breast cancer.

## 2. Materials and Methods

There are many players in the progression of breast tumors. However, to avoid too much complexity and to reduce the uncertainty of the model, we only considered very important players. The main cell types that we modeled were cancer cells, necrotic cells, T-cells, macrophages, dendritic cell, and adipocytes (Figure 1).

### 2.1. Interaction Network of Cells and Molecules—ODE Model

#### 2.1.1. T-Cells

In this model, T-cells are divided into four subgroups of naive, helper, cytotoxic, and regulatory T-cells. Naive T-cells, denoted by $T_{N}$, are not necessarily part of the tumor microenvironment, as they are usually activated within lymph nodes. We excluded them from the total number of cells in the microenvironment. Even though there are other methods, such as introducing non-linear terms in the ODEs to avoid unlimited exponential growth, making activation rates for other types of T-cells proportional to the number of naive T-cells was the most convenient way to create a controlled system given the complexity of our model. Thus, we summarize the equation for the dynamics of naive T-cells after deriving the equations of other types of T-cells. The variables $T_{h}$, $T_{C}$, and $T_{r}$, respectively, denote the numbers of activated T-helper cells, cytotoxic T-cells, and T-reg cells.

##### CD4+ Helper T-Cells ($T_{h}$)

CD4+ T-cells are activated by dendritic cells [16], HMGB1 [53], and IL-12 [23,54]. CD4+ T-cells’ phenotype expression is also promoted by estrogen [19]. On the other hand, regulatory T-cells [22] and IL-10 [55] inhibit CD4+ T-cells. Therefore, we modeled the dynamics of T-cells using the following equation:

(1) $$\begin{array}{cc} \frac{d[T_{h}]}{dt}= & \left(\lambda_{T_{h}H}\left[H\right]+\lambda_{T_{h}D}\left[D\right]+\lambda_{T_{h}IL_{12}}\left[IL_{12}\right]+\lambda_{T_{h}E}\left[E\right]\right)\left[T_{N}\right]\\ \; & -\left(\delta_{T_{h}T_{r}}\left[T_{r}\right]+\delta_{T_{h}IL_{10}}\left[IL_{10}\right]+\delta_{T_{h}}\right)\left[T_{h}\right]. \end{array}$$

##### Cytotoxic T-Cells ($T_{c}$)

Estrogen promotes the CD8+ T-cell phenotype’s expression [19,56]. Dendritic cells [18,57] and IL-12 activate naive CD8+ T-cells [23,54]. On the other hand, CD8+ T-cells’ function is suppressed by regulatory T-cells [23] and IL-10 [55]. Since natural killer (NK) cells also directly kill cancer cells, we assume this group includes both CD8+ T-cells and NK cells. Therefore, we modeled the dynamics of cytotoxic T-cells in the following way.

(2) $$\frac{d[T_{c}]}{dt}=\left(\lambda_{T_{c}E}\left[E\right]+\lambda_{T_{c}D}\left[D\right]+\lambda_{T_{c}IL_{12}}\left[IL_{12}\right]\right)\left[T_{N}\right]-\left(\delta_{T_{c}T_{r}}\left[T_{r}\right]+\delta_{T_{c}IL_{10}}\left[IL_{10}\right]+\delta_{T_{c}}\right)\left[T_{c}\right].$$

##### Regulatory T-Cells ($T_{r}$)

Dendritic cells stimulate the formation [17] and activation of regulatory T-cells [57]. Furthermore, estrogen enhances the actions of regulatory T-cells [19,58]. Hence, we used the following equation for the dynamics of T-reg cells.

(3) $$\frac{d[T_{r}]}{dt}=\left(\lambda_{T_{r}D}\left[D\right]+\lambda_{T_{r}E}\left[E\right]\right)\left[T_{N}\right]-\delta_{T_{r}}\left[T_{r}\right].$$

##### Naive T-Cells ($T_{N}$)

By combining the above-mentioned equations for the activation of naive T-cells and introducing independent naive T-cell production rate $A_{T_{N}}$, one can get the following equation for dynamics of naive T-cells:

(4) $$\begin{array}{ccc} \frac{d[T_{N}]}{dt} & = & A_{T_{N}}-\left(\lambda_{T_{h}H}\left[H\right]+\lambda_{T_{h}D}\left[D\right]+\lambda_{T_{h}IL_{12}}\left[IL_{12}\right]+\lambda_{T_{h}E}\left[E\right]\right)\left[T_{N}\right]\\ & - & \left(\lambda_{T_{c}E}\left[E\right]+\lambda_{T_{c}D}\left[D\right]+\lambda_{T_{c}IL_{12}}\left[IL_{12}\right]\right)\left[T_{N}\right]\\ & - & \left(\lambda_{T_{r}D}\left[D\right]+\lambda_{T_{r}E}\left[E\right]+\delta_{T_{N}}\right)\left[T_{N}\right]. \end{array}$$

#### 2.1.2. Dendritic Cells (*D*)

Dendritic cells are activated by cancer cells [23] and HMGB1 [59,60,61]. Moreover, estrogen enhances the metabolism, proliferation, differentiation, and functionality of dendritic cells [19], and multiple factors induced by cancer cells may promote natural dendritic cell death [57]. Denoting $A_{D_{N}}$ as the production rate of naive dendritic cells, we made the following system of equations for dynamics of naive dendritic cells ($D_{N}$) and activated dendritic cells (*D*):

(5) $$\begin{array}{ccc} \frac{d[D_{N}]}{dt} & = & A_{D_{N}}-\left(\lambda_{DC}\left[C\right]+\lambda_{DH}\left[H\right]+\lambda_{DE}\left[E\right]\right)\left[D_{N}\right]-\delta_{D_{N}}\left[D_{N}\right], \end{array}$$

(6) $$\begin{array}{ccc} \frac{d[D]}{dt} & = & \left(\lambda_{DC}\left[C\right]+\lambda_{DH}\left[H\right]+\lambda_{DE}\left[E\right]\right)\left[D_{N}\right]-\left(\delta_{DC}\left[C\right]+\delta_{D}\right)\left[D\right]. \end{array}$$

#### 2.1.3. Macrophages (*M*)

Since macrophages have many phenotypes and frequently change their phenotype, the breakdown of them into M1, M2, and other subsets would have tremendously complicated the model. Therefore, we modeled all activated macrophages as a single variable denoted by *M*. Tumor associated macrophages (TAMs) are activated by IL-10 [62,63]. IL-12 and IFN-γ activate M1 macrophages [24,62,64,65], and M2 macrophages are activated by IL-4 and IL-13, which are secreted by helper T-cells [62]. Moreover, estrogen exposure leads to alternative macrophage activation [19,58]. Denoting naive macrophages by $M_{N}$, activated macrophages by *M*, and the production rate of naive macrophages by $A_{M}$, we made the following system of equations for the dynamics of naive and activated macrophages.

(7) $$\begin{array}{cc} \frac{d[M_{N}]}{dt} & =A_{M}-\left(\lambda_{MIL_{10}}\left[IL_{10}\right]+\lambda_{MI_{\gamma }}\left[I_{\gamma }\right]+\lambda_{MIL_{12}}\left[IL_{12}\right]+\lambda_{MT_{h}}\left[T_{h}\right]+\lambda_{ME}\left[E\right]\right)\left[M_{N}\right]\\ \; & -\delta_{M_{N}}\left[M_{N}\right], \end{array}$$

(8) $$\begin{array}{cc} \frac{d[M]}{dt} & =\left(\lambda_{MIL_{10}}\left[IL_{10}\right]+\lambda_{MI_{\gamma }}\left[I_{\gamma }\right]+\lambda_{MIL_{12}}\left[IL_{12}\right]+\lambda_{MT_{h}}\left[T_{h}\right]+\lambda_{ME}\left[E\right]\right)\left[M_{N}\right]\\ \; & -\delta_{M}\left[M\right]. \end{array}$$

#### 2.1.4. Cancer Cells (*C*)

Since cancer cells proliferate at an abnormal rate, the proliferation of cancer cells is traditionally modeled by $\left[C\right](1-\left[C\right]/C_{0})$, where $C_{0}$ is the maximum capacity. In addition, IL-6 promotes the proliferation of cancer cells [66,67]. Additionally, adipocytes, releasing metabolic substrates, promote the proliferation of breast cancer cells [68]. On the other hand, activated CD8+ T-cells kill cancer cells [23,69], and IFN-γ initiates the elimination of cancer cells by inducing cell cycle arrest, apoptosis, and necroptosis [21]. The dynamics of cancer cells was modeled by the following equation.

(9) $$\frac{d[C]}{dt}=\left(\lambda_{C}+\lambda_{CIL_{6}}\left[IL_{6}\right]+\lambda_{CA}\left[A\right]\right)\left(1-\frac{\left[C\right]}{C_{0}}\right)\left[C\right]-\left(\delta_{CT_{c}}\left[T_{c}\right]+\delta_{CI_{\gamma }}\left[I_{\gamma }\right]+\delta_{C}\right)\left[C\right].$$

#### 2.1.5. Cancer Associated Adipocytes (*A*)

The direct crosstalk of cancer cells with tumor-surrounding stromal components, such as tumor-surrounding adipocytes, promotes tumor progression [70]. We modeled the proliferation of adipocytes similarly to the cancer cells’ proliferation.

(10) $$\frac{d[A]}{dt}=\lambda_{A}\left[A\right]\left(1-\frac{\left[A\right]}{A_{0}}\right)-\delta_{A}\left[A\right].$$

#### 2.1.6. Necrotic Cells (*N*)

Necrosis occurs when cells are under metabolic stress as their resources are being consumed by cancer cells [60]. Cells that go through the process of necrosis are denoted as necrotic cells. Since resources are limited in the cancer microenvironment, some cells will undergo necrotic cell death instead of other types of cell death [60,71]. Activated CD8+ T-cells kill cancer cells [23], and CD8+ cytotoxic T-cells produce IFN-γ, which then eliminates cancer cells [21]. Since a fraction of cancer cells can go through first becoming necrotic cells, the production rate of necrotic cells was modeled by the fraction ($\alpha_{NC}$) of dying cancer cells.

(11) $$\frac{d[N]}{dt}=\alpha_{NC}\left(\delta_{CI_{\gamma }}\left[I_{\gamma }\right]+\delta_{CT_{c}}\left[T_{c}\right]+\delta_{C}\right)\left[C\right]-\delta_{N}\left[N\right].$$

#### 2.1.7. Molecules

The dynamics of above mentioned molecules were modeled in the following way.

##### HMGB1 (*H*)

Damage-associated molecular pattern (DAMP) molecules are danger signals that promote inflammation and immune responses once released from dead or stressed cells [72]. The DAMP molecule, high-mobility group box 1 (HMGB1), exerts immune promoting activity by inducing angiogenesis, proliferation, and invasiveness of cancer cells via recruiting immune inflammatory cells [59]. HMGB1 is secreted by mature dendritic cells [59,60,73], necrotic cells [23,73,74], macrophages, [73,75,76], natural killer (NK) cells (which behave like cytotoxic T-cells) [73,77,78], and cancer cells [23,59,60].

For this reason, the dynamics of HMGB1 was modeled by the following equation.

(12) $$\frac{d[H]}{dt}=\lambda_{HD}\left[D\right]+\lambda_{HN}\left[N\right]+\lambda_{HM}\left[M\right]+\lambda_{HT_{c}}\left[T_{c}\right]+\lambda_{HC}\left[C\right]-\delta_{H}\left[H\right].$$

##### IL-12 ($IL_{12}$)

IL-12, which stimulates the growth and functions of T-cells, is involved in the differentiation of naive T-cells into helper T-cells. IL-12 is secreted by macrophages and dendritic cells [23,57,64]. Helper and cytotoxic T-cells also produce IL-12 [19]. We modeled the dynamics of IL-12 using the following equation.

(13) $$\frac{d[IL_{12}]}{dt}=\lambda_{IL_{12}M}\left[M\right]+\lambda_{IL_{12}D}\left[D\right]+\lambda_{IL_{12}T_{h}}\left[T_{h}\right]+\lambda_{IL_{12}T_{c}}\left[T_{c}\right]-\delta_{IL_{12}}\left[IL_{12}\right].$$

##### IL-10 ($IL_{10}$)

IL-10, which inhibits protective immune response (helper and cytotoxic T-cells), is produced by macrophages [62,79], dendritic cells [57,80,81], T-reg cells [55,82], CD4+ helper T-Cells [19,83], CD8+ cytotoxic T-cells [19,82], and cancer cells [17]. Therefore, the dynamics of IL-10 was modeled in the following way.

(14) $$\begin{array}{cc} \frac{d[IL_{10}]}{dt} & =\lambda_{IL_{10}M}\left[M\right]+\lambda_{IL_{10}D}\left[D\right]+\lambda_{IL_{10}T_{r}}\left[T_{r}\right]+\lambda_{IL_{10}T_{h}}\left[T_{h}\right]+\lambda_{IL_{10}T_{c}}\left[T_{c}\right]\\ \; & +\lambda_{IL_{10}C}\left[C\right]-\delta_{IL_{10}}\left[IL_{10}\right]. \end{array}$$

##### Estrogen (*E*)

Adipocytes are the primary producers of estrogen [84,85]. In breast tumors of postmenopausal women, estrogen can reach levels orders of magnitude greater than the low levels circulated in the body [85]. In general, adipose tissues in the breasts, brain tissues, osteoblasts, and other tissues locally produce estrogen, which circulates throughout the body. This amount of reproduced estrogen in the body depends on the pre-exiting existing amount. For this reason, we modeled the production rate of estrogen throughout the body using $\lambda_{E}\left[E\right]\left(1-\frac{\left[E\right]}{E_{0}}\right)$. This gave us the following equation for the dynamics of estrogen.

(15) $$\frac{d[E]}{dt}=\lambda_{EA}\left[A\right]+\lambda_{E}\left[E\right]\left(1-\frac{\left[E\right]}{E_{0}}\right)-\delta_{E}\left[E\right].$$

##### IFN-γ ($I_{\gamma }$)

CD8+ T-cells and CD4+ T-cells release IFN-γ [19,20,21]. Dendritic cells also secrete IFN-γ, but when exposed to estrogen, this production is increased [86]. Therefore, we modeled the dynamics of IFN-γ in the following way.

(16) $$\frac{d[I_{\gamma }]}{dt}=\lambda_{I_{\gamma }T_{c}}\left[T_{c}\right]+\lambda_{I_{\gamma }T_{h}}\left[T_{h}\right]+\lambda_{I_{\gamma }D}\left[E\right]\left[D\right]-\delta_{I_{\gamma }}\left[I_{\gamma }\right].$$

##### IL-6 ($IL_{6}$)

The important cytokine that leads to proliferation of cancer cells is IL-6 and is secreted by cancer associated adipocytes [64,66,67], macrophages [23,62,64,65,87], and dendritic cells [19,23,57].

(17) $$\frac{d[IL_{6}]}{dt}=\lambda_{IL_{6}A}\left[A\right]+\lambda_{IL_{6}M}\left[M\right]+\lambda_{IL_{6}D}\left[D\right]-\delta_{IL_{6}}\left[IL_{6}\right].$$

### 2.2. Data of the Model

#### 2.2.1. Breast Cancer Patients’ Data

There are some popular tumor deconvolution methods used to estimate the percentages of different immune cell types from the gene expression profiles of tumors. Among these methods, CIBERSORTx [88] has shown great performance in several studies [71,89,90,91]. In this study, we applied CIBERSORTx B-mode to gene expression profiles of primary tumors of breast cancer patients obtained from the Cancer Genome Atlas (TCGA) project of breast cancer (BRCA) [92] and the Molecular Taxonomy of Breast Cancer International Consortium (METABRIC) cohort [93]. There are 1904 primary breast tumor samples in the METABRIC microarray data downloaded from cBioPortal [94] and 1218 primary breast tumors with RSEM normalized RNA-seq data in $\log_{2}$ scale in the TCGA data downloaded from the University of California Santa Cruz (UCSC) Xena web portal [95]. Before performing CIBERSORTx B-mode on both datasets separately, we transformed TCGA data to the linear space and dropped samples of normal breast tissues. After estimation of cell proportions, we only considered cases with CIBERSORTx B-mode *p*-values <0.05 and continued our study with 2993 patients. We used expression values of genes encoding the molecules in the dynamical model and combined some immune cells frequencies to estimate the values of model’s variables as described in Table 1. Note that the genes’ expression levels in TCGA data were scaled depending on METABRIC data to eliminate the effect of different ranges in the two datasets on the results.

#### 2.2.2. Patient Data Analysis

**Figure 2:**
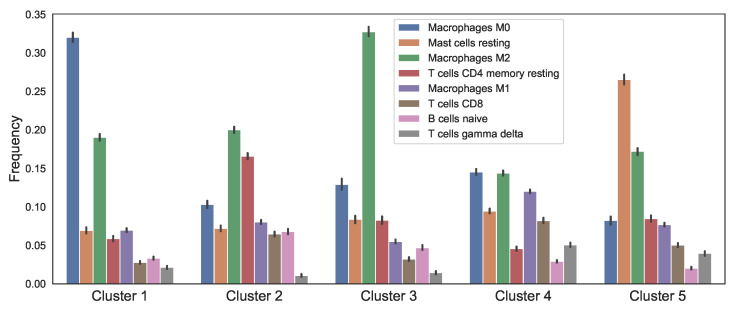
**Immune cell frequencies in each cluster.** Clusters were obtained by applying *K*-means clustering to the percentages of 22 immune cell types in breast tumors. The most variant cells among clusters are shown in this figure.

We grouped patients based on their estimated immune cell fractions using *K*-means clustering and determined the value of *K* using the elbow method. As a result, there were five distinct immune patterns of breast tumors. Figure 2 shows the average cell fractions in tumors of each cluster for the most variant immune cells among clusters, plus the standard deviation of each cluster as a bar.

Since the deconvolution method only provides us the percentage of each immune cell type in primary tumors, we used tumor weight for METABRIC data and tumor size for TCGA data, as described below, to estimate the numbers of immune cells, cancer cells, and necrotic cells in each tumor. For numerical stability, if the percentage of an immune cell that was used in the model was zero, it was substituted with 10% of the smallest positive cell fraction in the deconvolution data. We also excluded patients if their necrosis percentage or their tumor size in TCGA data, or the weight of their tumor in METABRIC data, were not available.

First, for simplicity, we assumed that the average number of cancer cells was twice the average number of total immune cells [37]. Therefore, using the necrosis percentage given in the TCGA data, the average ratio of immune cells to cancer cells to necrotic cells is approximately 0.3:0.6:0.1 in breast tumors. Additionally, the epithelial cell density has been reported as $4.5\times 10^{4}$ cells/cm${}^{3}$ in breast cancer [96]. We therefore choose the scaling factor $\alpha =4.5\times 10^{4}$ so that the average density of cancer cells across all patients was close to that value.

For each patient $P_{tcga}$ in TCGA data, the total cell number (${\mathrm{TCN}}_{tcga}$) was assumed to be proportional to the weight of the tumor:

$${\mathrm{TCN}}_{tcga}=\alpha \frac{\mathrm{tumor}\;\mathrm{weight}(P_{tcga})}{\frac{1}{K}\sum_{\mathrm{all}\;P_{tcga}}\mathrm{tumor}\;\mathrm{weight}\left(P_{tcga}\right)}$$

where *K* is the number of patients in TCGA data. For TCGA data, numbers of necrotic cells ($N_{\mathrm{tcga}}$), cancer cells ($C_{\mathrm{tcga}}$), and total immune cells (${\mathrm{TIC}}_{\mathrm{tcga}}$) were calculated using the given necrotic percentage ($N_{p}$) for each patient:

$$N_{\mathrm{tcga}}={\mathrm{TCN}}_{tcga}N_{p}\;\;,\;\;C_{\mathrm{tcga}}=\frac{2}{3}{\mathrm{TCN}}_{tcga}\left(1-N_{p}\right)\;\;\mathrm{and}\;\;{\mathrm{TIC}}_{\mathrm{tcga}}=0.5C_{\mathrm{tcga}}.$$

For METABRIC data, tumor size was used to calculate total cell number (${\mathrm{TCN}}_{meta}$) similarly to the TCGA data:

$${\mathrm{TCN}}_{meta}=\alpha \frac{\mathrm{tumor}\;\mathrm{size}(P_{meta})}{\frac{1}{K}\sum_{\mathrm{all}\;P_{meta}}\mathrm{tumor}\;\mathrm{size}\left(P_{meta}\right)}.$$

where *K* is the number of patients in the METABRIC data. Since the necrotic percentage was not available for METABRIC data, we used immune cells proportion from deconvolution data to calculate the total number of immune cells (${\mathrm{TIC}}_{\mathrm{meta}}$) for each patient ($P_{meta}$):

$${\mathrm{TIC}}_{\mathrm{meta}}=0.3\alpha \frac{\sum \mathrm{Immune}\;\mathrm{Cells}\;\mathrm{Ratio}(P_{meta})}{\frac{1}{K}\sum_{\mathrm{all}\;P_{meta}}\mathrm{Immune}\;\mathrm{Cells}\;\mathrm{Ratio}\left(P_{meta}\right)}\;\;\mathrm{so}\;\mathrm{that}$$

$$C_{\mathrm{meta}}=\frac{6}{7}\left({\mathrm{TCN}}_{meta}-{\mathrm{TIC}}_{\mathrm{meta}}\right)\;\;\mathrm{and}\;N_{\mathrm{meta}}=\frac{C_{\mathrm{meta}}}{6}.$$

Once we have the amount of all the cells and molecules we can extract useful information such as initial conditions (Table 2) and steady state values (Table 3) for each cluster.

#### 2.2.3. Parameter Estimation

We used 17 equations and 75 parameters in our model, which needed to be determined. We found the values of $\delta_{T_{N}},\;\delta_{T_{c}},\;\delta_{T_{h}},\;\delta_{T_{r}},\;\delta_{D},\;\delta_{M},\;\delta_{A},\;\delta_{H},\;\delta_{E},\;\delta_{IL_{6}},\;\delta_{IL_{10}},\;\delta_{IL_{12}}$ and $\delta_{I_{\gamma }}$ by finding their corresponding cell and molecule half-lives in the biological literature. For the rest of the parameters, we derived them so that the dynamics of all cells and molecules reached their steady state within our simulation time. In other words, for an ODE of the type $\frac{d\bar{X}}{dt}=F\left(\bar{X},\theta ,t\right)$, we solved:

(18) $$F(X^{\infty },\theta ,T)=0$$

for the parameter vector $\theta =\langle \theta_{1},\cdots ,\theta_{N}\rangle$, where *T* is the maximum simulation time and $X^{\infty }$ is the vector of steady-state values for the state variables given in Table 3. However, even with all of the known death rate parameters, we still had 62 more to determine and only 17 steady state equations of type (18). To circumvent this issue, we added some assumptions which were basically relationships between the parameters. These relationships were based on assuming some production rates or death rates within the same ODE would be more effective than the rest.The relationship formulations were created by the authors to make sure we got a positive set of parameters. For more technical details of the parameter estimation process, please refer to Appendix A.1.

#### 2.2.4. Sensitivity Analysis

As we mentioned, because of the lack of biological information about many of parameters’ values, we made several assumptions about these parameters given in Appendix A.1. To remedy this important limitation of the model, we perform a global gradient-based sensitivity analysis by changing each of these assumptions 5000 times, and obtaining a new set of parameter values with each new assumption. Since the number of parameter values (number of assumptions × number of variations =38×5000=190,000) still is a finite number, this limitation of the model must be considered when the results of the model are used. For better numerical stability, we performed the sensitivity analysis using the non-dimensionalized system (see Appendix A.2 for more details).

Generally, the level of sensitivity of a variable *X*, to its vector of parameters $\theta =\langle \theta_{1},\cdots ,\theta_{N}\rangle$ for an ODE of the type $\frac{d\bar{X}}{dt}=F\left(\bar{X},\theta ,t\right)$, is calculated by:

(19) $$s_{i}=\frac{d\bar{X}}{d\theta_{i}},\;\mathrm{for}\;i=1,\cdots ,N.$$

We calculated the sensitivity of cancer cells and the total number of cells for each parameter $\theta_{i}$ at the steady state. In other words, for the steady state values ${\bar{X}}^{*}$ the sensitivity vector $s=\langle s_{1},\cdots ,s_{N}\rangle$ was obtained by differentiating $F({\bar{X}}^{*},\theta )=0$ with respect to θ. We got this analytical formula:

(20) $$s=\frac{d{\bar{X}}^{*}}{d\theta }=-{\left(\nabla F({\bar{X}}^{*},\theta )\right)}^{-1}\left(\frac{\partial F({\bar{X}}^{*},\theta )}{\partial \theta }\right),$$

where ${\left(\nabla F({\bar{X}}^{*},\theta )\right)}^{-1}$ is the numerical inverse of Jacobian of F with respect to $\bar{X}$.

To calculate the global sensitivity, we followed the following steps:

- First, we define a local sensitivity measure for each parameter $\theta_{i}$ in the neighborhood $\Omega^{k}\left(\theta_{i}\right)$ as
  (21) $$S_{i}^{k}=\int_{\Omega^{k}\left(\theta_{i}\right)}s_{i}\left(\theta \right)d\theta .$$
  These neighborhoods were acquired by varying assumptions (A1)–(A22) by scaling factors 0.01 to 100. The integration was carried out numerically using sparse grid points [97,98].
- Second, we found weights for the aforementioned neighborhoods. Scaling each assumption provides us with a new set of parameters. The weights were then determined by calculating the distance of each resulting parameter set to a fixed base parameter set. We assigned higher weights to the parameters that were closer to the base values. We denote each weight by $w_{i}^{k}$ for i=1,⋯,N and k=1,⋯,K corresponding to the parameter and its neighborhood, respectively.
- Finally, we obtained the global sensitivity level $S_{i}$ to each parameter $\theta_{i}$ by
  (22) $$S_{i}=\sum_{k=1}^{K}w_{i}^{k}S_{i}^{k}.$$

## 3. Results

### 3.1. Data Analysis of the Clusters

**Figure 3:**
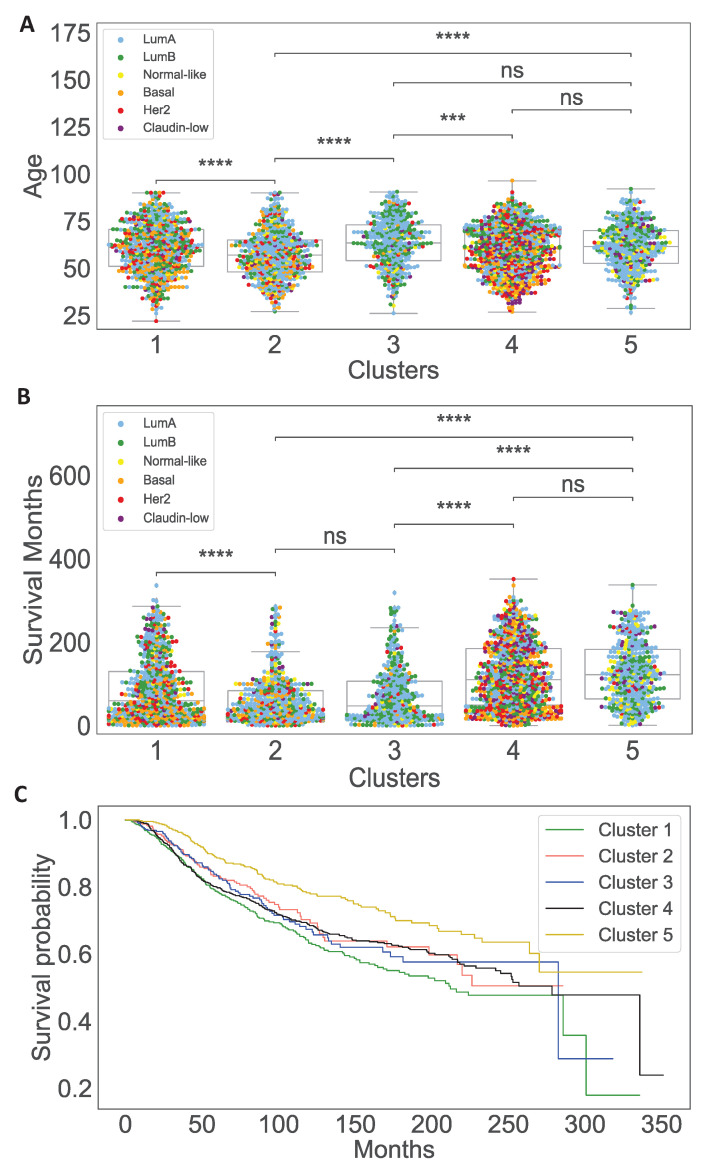
**More Clinical features of each cluster.** Subfigures (**A**,**B**), respectively, show box plots of patients’ age at diagnosis and survival months in each cluster. Subfigure (**C**) demonstrates Kaplan–Meier curves of overall survival probability across five clusters. Asterisks in the figures show the significance levels with Mann–Whitney-Wilcoxon (MWW) statistical test where, ns: no significance, ***: 0.0001<p≤0.001, ****: p≤0.0001.

**Figure 4:**
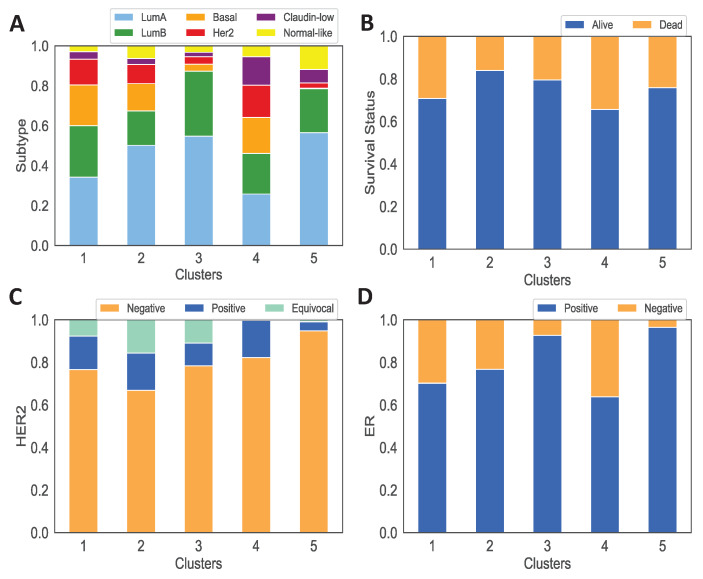
**Clinical features of each cluster.** Subfigures (**A**–**D**) show the percentage of patients with different subtypes of breast cancer, survival status, HER2 status, and ER status, respectively.

We also use TCGA and METABRIC clinical data to analyze the clinical features of each cluster and their associations with tumor immune microenvironment and dynamics. We see that cluster 2 and cluster 3, respectively, include a significant high number of the youngest and oldest patients compared to the other clusters (Figure 3A). We also observe that cluster 5 has the highest percentage of tumors with estrogen receptor (ER+) and human epidermal growth factor receptor 2 negative (HER2-) tumors, and ultimately a high number of LumA, LumB and normal-like tumors. On the other hand, clusters 1 and 4 have a higher percentage of aggressive subtypes such as HER2-enriched and Basal than other clusters, which explains having the highest percentage of tumors without estrogen receptor (ER-) in these clusters (Figure 4A,C,D). In addition, survival probabilities of cluster 1 and cluster 4 are lower than other clusters while cluster 5, which has more non-aggressive tumor, has the best survival results (Figure 3C). Note that survival status of the patients is given as alive or dead in TCGA data while in METABRIC data dead patients are separated into two categories ‘died of other causes’ and ‘died of disease’ so we translate ‘died of other causes’ into alive status. For this reason, we see survival probability of cluster 3 is lower in Figure 3C while the percentage of alive patients in cluster 3 is higher (Figure 4B).

### 3.2. Dynamics of the Breast Cancer Microenvironment

We find the dynamics of each variable involved in tumor microenvironment by solving the ODEs (1)–(17) with parameters acquired from the steady state assumptions for each cluster (Appendix A.1). We derive the dynamics of all of the cells and molecules based on the non-dimensional parameters in Table A4, half-lives and estimated death rates from Table A3, and initial conditions acquired from the tumors with the smallest size in each cluster (Table 2) and their steady state values in Table 3. We also plot the changes in total cells. We define total cells as:

(23) $$\mathrm{Total}\;\mathrm{Cells}=T_{h}+T_{c}+T_{r}+D_{N}+D+0.2M_{N}+M+C+N+A$$

We exclude $T_{N}$ since they are mainly found in the circulation and lymphatic system [99]. The rest of the T-cells are known as tumor infiltrating T-cells and can be found abundantly in tumors’ microenvironment. Dendritic cells primarily get activated inside of the tumor and cancer cells, necrotic cells and adipocytes are the other main components of the breast tumor, [12]. Finally, since most of naive macrophages polarize into M1 (anti-tumor) and M2 (pro-tumor) phenotypes inside of the tumor we consider a 20% factor for $M_{N}$,^100^].

**Figure 5:**
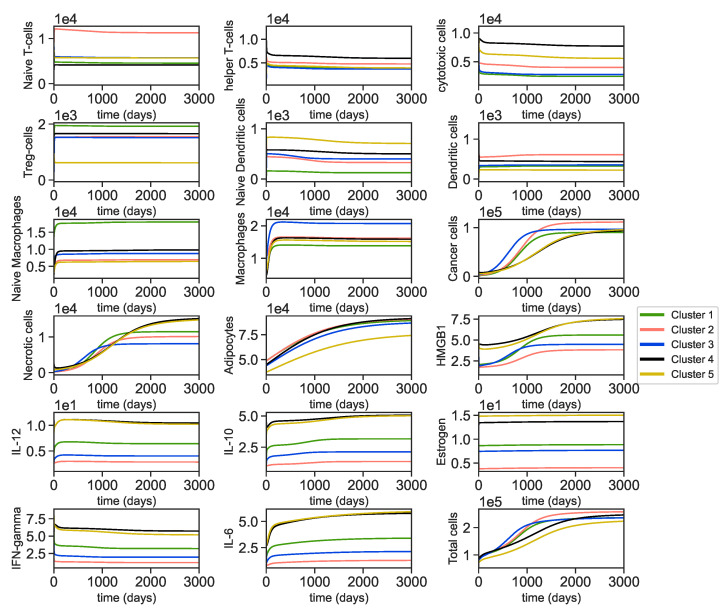
**Dynamics of all variables.** Dynamics of variables of the model over 3000 days. The different color lines describe the dynamics of different clusters.

The dynamics of cells given in Figure 5 shows that for all clusters except cluster 2, the naive T-cells increase from their initial values and reach the steady state quickly, while the naive T-cells in cluster 2 increase and then decrease in a slow manner to reach the steady state. Helper T-cells increase from their initial condition and then decrease to reach the steady state quickly. Cytotoxic T-cells also follow the same pattern. T-reg cells reach the steady state quickly and remain constant at the steady state. While the naive dendritic cells start with a higher population initially and decrease eventually to reach the steady state, mature dendritic cells start with a low initial population and increase eventually to reach their steady states. This is because mature dendritic cells are derived from naive dendritic cells. On the other hand, both naive macrophages and macrophages increases within a short amount of time before reaching their steady states.

Cancer cells dynamics show an exponential growth until they reach the steady state. Since cancer cells go through necrosis, the necrotic cells’ growth behaves similarly. Adipocytes activation increases over time until they reach the steady state for all clusters. The amount of estrogen also increases from their initial conditions in each cluster as it is produced by adipocytes.

The initial concentration of HMGB1 in clusters 4 and 5 is higher than other clusters and stay higher in their steady state. The general trend for HMGB1 is increasing for all clusters before it reaches the steady state.

Although the concentration of IL-12 increases at the beginning, it lowers from the maximum concentration to reach the steady state. IL-10 and IL-6 increase from their initial value to reach the steady state. IFN-γ increases first then decreases over time as the cancer cells increase.

Due to the shear abundance of cancer cells and adipocytes compared to other cells, the total number of cells is more significantly affected by these cells. We can see that cluster 5 has one of the lowest cancer cells and the lowest adipocytes population, so the total cells population is the lowest in cluster 5. Additionally, cluster 2 has the highest cancer cells and one of the highest adipocytes in its steady state. As a result, total cells dynamics is the highest for cluster 2. Since total cells population is proportional to tumor sizes, it can be inferred that cluster 2 has larger tumors than the other clusters.

While cluster 3 has the fastest cancer growth, cluster 2 has the highest cancer population at the steady state among the other clusters. Additionally, cluster 2 has the highest population of dendritic cells that inhibit cancer cells and being activated by cancer cells. As dendritic cells are also activated by estrogen and HMGB1, their concentrations are the lowest in cluster 2. Thus, the higher population of dendritic cells may be mostly due to cancer cells’ activation. Note, cluster 2 includes the primary tumors of the youngest patients (Figure 3B) and aggressive tumors (Figure 4A), and it is known that aggressive breast tumor in younger patients grow faster [101].

On the other hand, cluster 4 and 5 have the slowest cancer growth, while cluster 1 has the lowest cancer population at the steady state compared to other clusters. Interestingly, the amount of immune cells that are known to be correlated with good prognoses such as cytotoxic T-cells and helper T-cells are the lowest in the cluster 1 which might cause making a wrong prediction at first glance. Thus, it is more important to consider interactions between the immune cells and cancer cells than just looking at their quantities to understand cancer prognosis.

Cluster 5 has the best survival probability according to Figure 3C. The population of adipocytes is the lowest in cluster 5 compared to the other clusters. Dendritic cells population, and the concentration of HMGB1 and estrogen are very low. Furthermore, cluster 5 has one of the lowest cancer cells population at the steady state. Furthermore, Figure 4C,D illustrate that cluster 5 has the lowest HER2-positive and ER-positive patients that are indicators of a better prognoses.

Although dynamical results show a lower cancer growth for cluster 1 and 4, clinical data analysis shows that cluster 1 and 4 have the lowest survival probability among the other clusters as illustrated in Figure 3C. This can be due to the fact that cluster 1 and 4 have one of the highest population of adipocytes at the steady state and thus providing more resources for cancer cells. Furthermore, based on Figure 4A these clusters have the highest percentage of aggressive tumors.

### 3.3. Sensitivity Analysis

After finding the base parameters from the steady state assumptions in Appendix A.1 and the steady state values given in Table 3, we carry out the global sensitivity analysis mentioned in Section 2.2.4.

**Figure 6:**
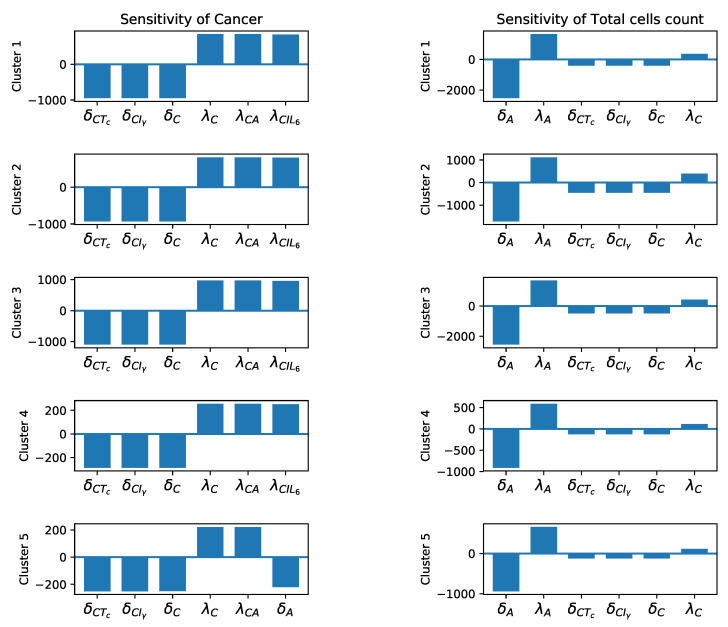
**Sensitivity analysis.** Sensitivity level of the most sensitive parameters for cancer and total cell population, respectively.

**Figure 7:**
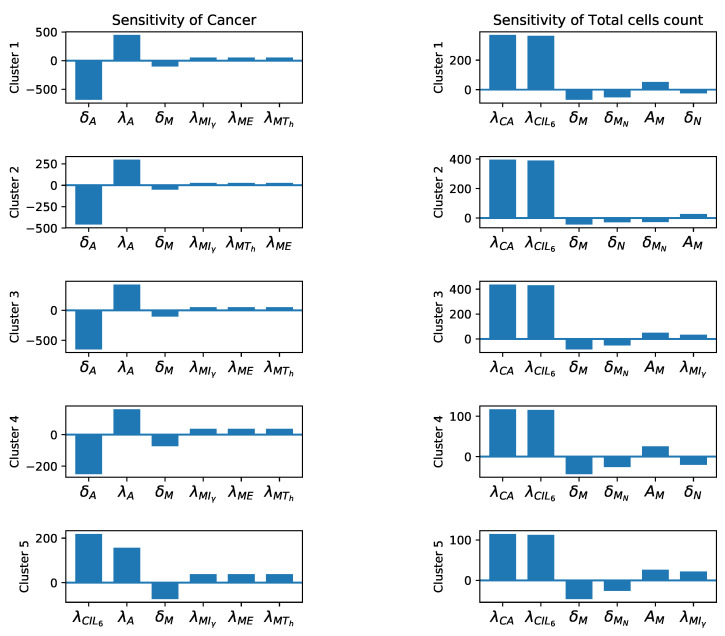
**Sensitivity analysis.** Other sensitive parameters for cancer and total cell population.

We investigate the global sensitivity of cancer cells and the total number of cells (Equation (23)) to each parameter. The results are given in Figure 6 and Figure 7. As expected, we see the cancer population is the most sensitive to cancer related parameters (Figure 6), and since total cells includes high number of cancer cells (Figure 5), they are also sensitive to these parameters (Figure 6 and Figure 7). In addition, total cells and cancer populations in all clusters are significantly sensitive to $\delta_{A}$ and $\lambda_{A}$. This is reasonable, since all clusters have high numbers of adipocytes that directly contribute to cancer proliferation Equation (9). All of the sensitivity plots imply that less decay and more production of adipocytes lead to larger number of cancer and total cells.

Finally, cancer population in all clusters are directly sensitive to macrophage promoting parameters and reversely sensitive to the macrophage decay rates. We have modeled both macrophage phenotypes (M1 and M2) together. However, in all the clusters the frequency of M2 (pro-tumor) phenotype is more dominant (Figure 2). Therefore, more macrophages should result in more cancer cells and consequently higher number of total cells. The presence of $\delta_{M_{N}}$ as a sensitive parameter in Figure 7 can be justified in the same way as a significant number of naive macrophages turn into activated macrophages. Moreover, in clusters 1, 2, and 4, we see that clearance of necrotic cells has an opposite effect on the total number of cells which is reasonable, given the presence of the necrotic cells in the tumor microenvironment. In clusters 3 and 5, $\delta_{N}$ is replaced by $\lambda_{MI_{\gamma }}$, because cluster 3 has a very high number of macrophages and cluster 5 has a high level of $I_{\gamma }$, Figure 5.

### 3.4. Dynamics with Varying Assumptions

We investigate the effect of changing parameters’ assumptions on cancer dynamics in clusters 1–5. To avoid cluttering, we only target assumptions that incorporate sensitive parameters from the previous section, and we scale them by factors of 0.2 and 5. These scaled assumptions will produce new sets of parameters for each cluster leading to new dynamics. Furthermore, we locally perturb all the sensitive parameters from Section 3.3 to acquire a 10% variation region for cancer dynamics.

Notice that some sensitive parameters are either solely obtained from ODEs’ steady states or taken from the literature and are not explicitly included in these assumptions. Therefore, we only scale the following assumptions by 1.0, 0.2 and 5.0:

(24) $$\begin{array}{cc} \mathrm{Scale}\times \delta_{CT_{c}}\left[T_{c}^{\max}\right] & =6\delta_{CI_{\gamma }}\left[I_{\gamma }^{\max}\right] \end{array}$$

(25) $$\begin{array}{cc} \mathrm{Scale}\times \delta_{C} & =6\delta_{CI_{\gamma }}\left[I_{\gamma }^{\max}\right] \end{array}$$

(26) $$\begin{array}{cc} \mathrm{Scale}\times 2\lambda_{CA}\left[A^{\max}\right] & =\lambda_{CIL_{6}}\left[IL_{6}^{\max}\right] \end{array}$$

(27) $$\begin{array}{cc} \mathrm{Scale}\times \lambda_{MI_{\gamma }}\left[I_{\gamma }^{\max}\right] & =\lambda_{MIL_{10}}\left[IL_{10}^{\max}\right] \end{array}$$

(28) $$\begin{array}{cc} \mathrm{Scale}\times \lambda_{ME}\left[E^{\max}\right] & =\lambda_{MIL_{10}}\left[IL_{10}^{\max}\right] \end{array}$$

(29) $$\begin{array}{cc} \mathrm{Scale}\times \lambda_{MT_{h}}\left[T_{h}^{\max}\right] & =\lambda_{MIL_{10}}\left[IL_{10}^{\max}\right] \end{array}$$

**Figure 8:**
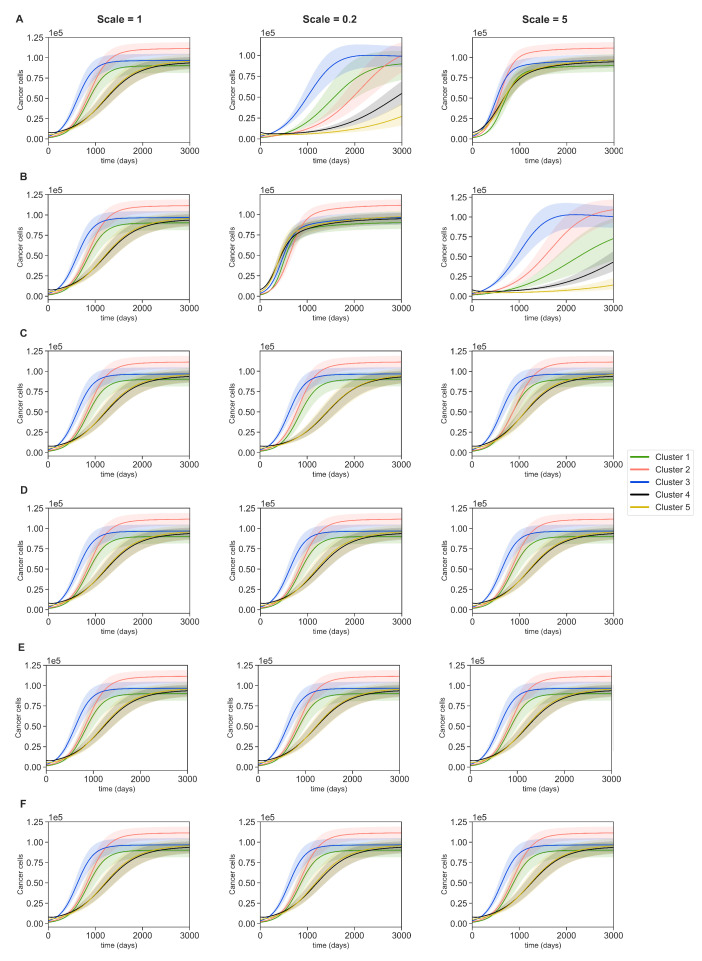
**Dynamics of cancer after the assumptions of the sensitive parameters were modified.** Subfigures (**A**–**F**) present dynamics after scaling the assumptions (24)–(29), respectively. The transparent region was the result of 10% perturbation of all the sensitive parameters from Section 3.3.

The results are shown in Figure 8. We can see that scaling the assumptions (24) and (25) had the largest effect on the dynamics of cancer cells across the five clusters. On the other hand, assumptions (26)–(29) negligibly affect the time of reaching steady state in clusters 4 and 5 when they are up-scaled. The assumption (24) delayed the time of reaching steady states for all clusters while it was scaled down and sped it up when it was scaled up. Clusters 2, 4, and 5 were the most affected in the case shown in Figure 8A; and clusters 1, 2, 4, and 5 were the most affected in the case shown in Figure 8B. Below is a comparison between the values of three crucial parameters for Figure 8A.

According to Table 4, downscaling the assumption (24) increased the value of $\delta_{CT_{c}}$ and decreased the values of $\delta_{CI_{\gamma }}$ and $\delta_{C}$. This is because both $\delta_{C}$ and $\delta_{CT_{c}}$ are related to each other via $\delta_{CI_{\gamma }}$. Since clusters 2, 4, and 5 had the highest levels of cytotoxic cells, increasing cytotoxic cells’ cancer inhibition effect ($\delta_{CT_{c}}$) remarkably decreased the number of cancer cells. Similarly, we got Table 5 when scaling the assumption (25). In this case, upscaling the assumption (25) decreased the value of $\delta_{C}$ and increased the values of $\delta_{CI_{\gamma }}$ and $\delta_{CT_{c}}$. For the same reason, cancer saturation in clusters 2, 4, and 5 underwent a delay. Moreover, cluster 1 which has a low level of cytotoxic cells but a reasonable level of IFN-γ can more effectively remove cancer cells with this combination of parameter values.

None of the cases above completely neutralized the tumor. Even in the extreme cases, such as clusters 4 and 5 in Figure 8A,B, the cancer population growth was just delayed and would reach its steady state in later times. However, the mentioned parameters can cause significant delays in cancer growth, especially in clusters 1 and 4, whose patients had low survival probabilities. Hence, targeting cells and molecules corresponding to these parameters can be crucial in cancer treatments.

To even further investigate the parameters involved in cancer ODE and have more flexibility in finding scenarios in which cancer completely vanishes, we carried out a bifurcation analysis (see Appendix A.6). We assumed that $\left[IL6\right],\left[A\right],\left[T_{c}\right]$ and $\left[I_{\gamma }\right]$ were at their steady state values for each cluster. Next, we chose one parameter as the bifurcation variable and let the rest of them take their estimated values from Table A4 for each cluster.

Our results show that three parameters from Table 4 and Table 5 gave non-zero equilibria for cancer for very small values, and they caused cancer to vanish for a large range. This is in-line with how sensitive the dynamics results were to these parameters. On the other hand, parameters such as $\lambda_{C}$, $\lambda_{CIL_{6}}$, and $\lambda_{CA}$ contribute to larger steady state values as they get larger. Saliently, cancer in clusters 1–5 vanished for very small values of $\lambda_{C}$, while small values of $\lambda_{CA}$ could significantly decrease the steady state population of cancer only in cluster 5. This result is very interesting, because cluster 5 had the highest survival rate (Figure 3), the highest number of LumA subtypes (Figure 4), and the lowest adipocyte population (Figure 5). For more details, see Appendix A.6.

### 3.5. Dynamics with Different Initial Conditions

We investigated the effects of different initial conditions on the dynamics of each cluster. We varied the initial conditions by extracting individual patient data from each cluster. Due to the large number of patients, we plotted the dynamics of tumors with the number of cancer cells below the 40th percentile as the initial conditions. We observe that the dynamics did not change regardless of different initial conditions. All the patients from one cluster converged to the same steady state, no matter whether they started from higher or lower initial quantities (Figure ). This is reasonable, since the parameters were derived based on the steady state assumptions for each cluster.

We also looked at the dynamics of small tumors in one cluster converging on a large tumor at the steady state in another cluster. We observe that even when the initial conditions were not from the same cluster as the parameters’ values, the dynamics of tumors quickly converged on the dynamics of tumors in the cluster of the steady state. This testifies that the impact of parameters on dynamics is more significant than the initial conditions (Appendix A.5)s.

## 4. Discussion

Understanding cancer mechanisms and components in in vitro and in vivo studies is time consuming and does not provide comprehensive results to explain cancer complexity, since each cancer component is studied one at a time [102]. With the advancements in tumor deconvolution methods and the availability of more data [103], the data-driven mathematical models have become popular for exploring the complexity of the system more effectively. In this study, we developed a mathematical model that explored the characteristic differences of breast tumors based on their tumor microenvironments. This model allowed us to understand tumor progression in more detail.

The mathematical results showed that tumor growth in each cluster demonstrates unique interactions with its tumor microenvironment. For example, as the number of cancer cells increases, the numbers of cytotoxic cells, helper T-cells, and IFN-γ increase at the beginning and then decrease to reach a steady state. Furthermore, while regulatory T-cells in clusters 1, 2, and 3 reached the steady state very early and remained constant throughout the time, in clusters 4 and 5, they increased very early and then decreased to reach the steady state. Importantly, it has been found that interactions among immune cells can impact the immunological response in various cancer types [104,105]. Our results also show that it is crucial to investigate the interactions among immune cells rather than simply looking at the quantity of a certain immune cell type to make appropriate prognostic predictions for breast cancer patients. The results also show that some of the variables, such as $T_{c}$, $T_{h}$, *D*, *E*, and $I_{\gamma }$, stayed approximately constant over time. Hence, one might simplify the model by removing the ODEs of these variables and assuming their steady state values in the remaining equations.

When we compare dynamical results with clinical information of the clusters, we can see that cluster 5, which has the best survival probability and one of the most non-aggressive tumor types (Figure 3C), had some of the lowest tumor growth and had a smaller cancer population at the steady state (Figure 5). Furthermore, we want to clarify that while cluster 3 contained mostly LumA and LumB breast tumors that tend to have better prognosis and better survival times compared to other subtypes of breast tumor, the survival probability of this cluster is low. This is because cluster 3 significantly consisted of the oldest patients compared to the other clusters (Figure 3A). In addition, while cluster 1 included more aggressive tumors than cluster 2, tumor growth in cluster 2 was the highest at the steady state. This might be due to the fact that cluster 2 had the youngest patients (Figure 3A), and aggressive breast tumors in young patients tend to grow more rapidly than ones in older women [101].

Having a large number of unknown parameters and difficulties in deriving their values are the main challenges in mathematical modeling of cancer. In this study, we derived some parameter values from experimental studies published in the literature, and we calculated others using the steady state assumptions and maximum values of the variables, using a similar method given in recent studies [71,106]. We estimated parameters uniquely for each group of patients with different immune profiles, because many studies have demonstrated that patients with different immune compositions show different prognoses and respond differently to therapy [107,108,109]. Thus, having separate parameters for each cluster allowed us to see the effects of immune cells on tumor growth more accurately.

**Figure A3:**
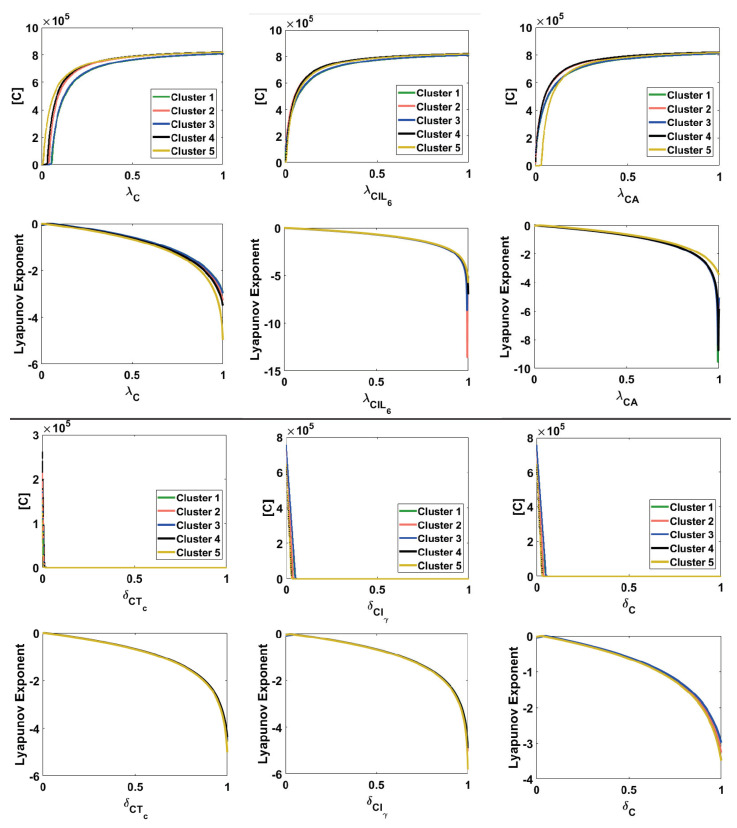
**Bifurcation and Lyapunov exponent diagrams for cancer parameters:** Subfigures show the bifurcation on top of the corresponding Lyapunov diagrams for clusters 1–5. Bifurcation was done for parameters $\lambda_{C}$, $\lambda_{CIL_{6}}$, $\lambda_{CA}$, $\delta_{CT_{c}}$, $\delta_{CI_{\gamma }}$, and $\delta_{C}$ from the cancer ODE (9).

In addition, we performed a global gradient-based sensitivity analysis to investigate the effects of each parameter on the dynamical system. The results of sensitivity and bifurcation analyses provided interesting insights about the biological mechanism, especially the importance of IFN-γ in controlling cancer growth (Figure 8 and Figure A3). We saw that the cancer population is primarily sensitive to parameters that directly promote or inhibit cancer cells, and secondarily, to parameters which promote or inhibit macrophages. In other words, cancer dynamics positively react to an increase in macrophages. It is reported that high levels of tumor associated macrophages are correlated with worse patient prognosis [100]. Moreover, to acquire patient specific parameters, we had to devise some mathematical assumptions about the parameters of our system. These assumptions were mostly mathematical artifacts ensuring nonzero parameter values. We chose the assumptions which involved the sensitive parameters in our sensitivity analysis and investigated the effect of scaling these assumptions to see how the cancer dynamics would change. We found out that we could delay cancer recovery time through certain assumptions. More specifically, we saw that the parameter $\delta_{CI_{\gamma }}$ was involved in the most interesting cases. In these cases, we saw a significant delay in cancer saturation time. Furthermore, according to our bifurcation analysis (see Appendix A.6), these parameters have a large range, which led to a vanishing cancer population that could translate into promising treatment options. The therapeutic potential of IFN-γ in later stages of cancer has been observed by researchers [110,111]. It is known that IFN-γ can directly inhibit breast cancer by restoring ICI-induced apoptosis in breast cancer cells that have acquired resistance to this antiestrogen [112], and the combination of anti-growth factor receptor MAb and cytokines such as IFN-γ may be useful in the treatment of breast cancer [113]. On the other hand, IFN-γ can increase the cytotoxicity of T-cells, leading to more effective inhibition of cancer cells [114,115]. Our bifurcation results also showed that controlling the production of cancer via adipocytes can significantly decrease the cancer population at the steady state for LumA subtypes. It has been observed that an excessive amount of adipocytes is associated with advanced T stages of the triple-negative and LumA subtypes, and the modulation of adipocytes can provide novel therapy targets for breast cancer [116]. Besides these, we also investigated how a dynamical system would be affected if we used different initial conditions for each cluster, and we saw that the parameter values that were used in each cluster were more important than the initial conditions for determining the dynamics.

The results of this study should be used by considering the limitations of this study. While the use of time course data would be ideal to obtain parameters for each cluster, the availability of such data is limited. To combat this challenge, we used large amounts of tumor data in each cluster as the steady state values, to estimate parameters. Despite this limitation due to the lack of time course data, our model still presented essential information about the progression of breast tumors, and our study can be used to develop new models using only gene expression data of the patients. Moreover, one might utilize different approaches, such as applying partial differential equations, to investigate tumor growth in space and time, or utilize other parameter fitting algorithms [117,118,119,120] to obtain more accurate dynamical systems. Lastly, it has been shown that treatment options in breast cancer have significant effects on the immune system. This model can be used for inferring treatment strategies in breast cancer by adding the interactions between the effects of the treatments on the tumor microenvironment.

## Figures and Tables

**Table 1:** **Patient data correspondence with variables.** Correspondence among the model variables and the gene expression data of the primary tumors and deconvolution results.

Variable	Name	Data Used
$T_{N}$	Naive T-cells	Combination of CD4 naive and memory resting T-cells and resting NK cells
$T_{h}$	Helper T-cells	Combination of memory activated CD4 T-cells and follicular helper T-cells
$T_{C}$	Cytotoxic cells	Combination of CD8 T-cells and activated NK cells
$T_{r}$	Regulatory T-cells	Regulatory T-cells
$D_{N}$	Naive dendritic cells	Naive dendritic cells
*D*	Activated dendritic cells	Activated dendritic cells
$M_{N}$	Naive Macrophages	Combination of Macrophages M0 and Monocytes
*M*	Macrophages	Combination of M1 and M2 Macrophages
*C*	Cancer cells	Estimated
*N*	Necrotic cells	Estimated
*A*	Cancer Associated Adipocytes	Assumed to be twice the total number of immune cells
*H*	HMGB1	HMGB1 gene expression
$IL_{12}$	IL-12	IL12A and IL12B gene expressions
$IL_{10}$	IL-10	IL10 gene expression
*E*	Estrogen	ESR1 and ESR2 gene expressions
$I_{\gamma }$	IFN-γ	IFNG gene expressions
$IL_{6}$	IL-6	IL6 gene expression

**Table 2:** **Initial conditions of the model variables.** Values of the initial conditions were obtained from the averages of the smallest tumors in METABRIC and TCGA data.

Cluster	$T_{N}$	$T_{h}$	$T_{C}$	$T_{r}$	$D_{N}$	D	$M_{N}$	M	C
1	$2.62\times 10^{2}$	$1.93\times 10^{3}$	$2.94\times 10^{3}$	$5.55\times 10^{2}$	$9.90\times 10^{-3}$	$9.90\times 10^{-3}$	$1.34\times 10^{4}$	$5.37\times 10^{3}$	$1.56\times 10^{3}$
2	$3.78\times 10^{3}$	$2.16\times 10^{3}$	$2.95\times 10^{3}$	$5.04\times 10^{2}$	$1.07\times 10^{3}$	$9.83\times 10^{3}$	$2.63\times 10^{3}$	$1.12\times 10^{4}$	$2.31\times 10^{3}$
3	$2.93\times 10^{3}$	$1.15\times 10^{3}$	$1.39\times 10^{3}$	$3.68\times 10^{1}$	3.22	$3.62\times 10^{-2}$	$6.75\times 10^{3}$	$9.67\times 10^{3}$	$3.53\times 10^{3}$
4	$4.78\times 10^{3}$	$4.60\times 10^{3}$	$2.76\times 10^{3}$	$1.66\times 10^{3}$	$3.51\times 10^{2}$	$1.34\times 10^{3}$	$2.29\times 10^{3}$	$4.69\times 10^{3}$	$7.96\times 10^{3}$
5	$3.78\times 10^{3}$	$1.33\times 10^{3}$	$3.11\times 10^{3}$	$1.05\times 10^{3}$	$5.16\times 10^{2}$	$1.03\times 10^{-2}$	$2.85\times 10^{3}$	$6.07\times 10^{3}$	$5.57\times 10^{3}$
	N	A	H	${\mathrm{IL}}_{12}$	${\mathrm{IL}}_{10}$	E	$I_{\gamma }$	${\mathrm{IL}}_{6}$	
1	$1.21\times 10^{2}$	$4.89\times 10^{4}$	5.06	5.82	2.90	7.62	3.05	3.67	
2	$3.16\times 10^{2}$	$4.86\times 10^{4}$	5.01	6.40	2.93	8.76	2.93	3.02	
3	$3.11\times 10^{2}$	$4.39\times 10^{4}$	5.29	5.25	2.79	9.16	2.81	4.27	
4	$2.71\times 10^{3}$	$4.49\times 10^{4}$	5.29	6.88	3.27	6.38	4.10	3.40	
5	$3.73\times 10^{2}$	$3.74\times 10^{4}$	6.16	5.67	2.66	8.63	2.70	3.03	

**Table 3:** **Steady state values of the model variables.** Large tumors in each cluster were grouped, and their average values were found for each variable, to be used in the parameter estimation.

Cluster	$T_{N}^{\infty }$	$T_{h}^{\infty }$	$T_{C}^{\infty }$	$T_{r}^{\infty }$	$D_{N}^{\infty }$	$D^{\infty }$	$M_{N}^{\infty }$	$M^{\infty }$	$C^{\infty }$
1	$4.55\times 10^{3}$	$3.87\times 10^{3}$	$2.44\times 10^{3}$	$1.92\times 10^{3}$	$1.26\times 10^{2}$	$3.28\times 10^{2}$	$1.80\times 10^{4}$	$1.39\times 10^{4}$	$9.03\times 10^{4}$
2	$1.13\times 10^{4}$	$4.77\times 10^{3}$	$4.02\times 10^{3}$	$1.55\times 10^{3}$	$3.27\times 10^{2}$	$6.10\times 10^{2}$	$6.96\times 10^{3}$	$1.62\times 10^{4}$	$1.12\times 10^{5}$
3	$5.73\times 10^{3}$	$3.70\times 10^{3}$	$2.79\times 10^{3}$	$1.51\times 10^{3}$	$4.00\times 10^{2}$	$3.52\times 10^{2}$	$8.77\times 10^{3}$	$2.07\times 10^{4}$	$9.68\times 10^{4}$
4	$4.14\times 10^{3}$	$5.95\times 10^{3}$	$7.71\times 10^{3}$	$1.66\times 10^{3}$	$4.99\times 10^{2}$	$4.37\times 10^{2}$	$9.81\times 10^{3}$	$1.59\times 10^{4}$	$9.54\times 10^{4}$
5	$5.69\times 10^{3}$	$3.91\times 10^{3}$	$5.56\times 10^{3}$	$6.16\times 10^{2}$	$7.04\times 10^{2}$	$2.22\times 10^{2}$	$6.45\times 10^{3}$	$1.52\times 10^{4}$	$9.90\times 10^{4}$
	$N^{\infty }$	$A^{\infty }$	$H^{\infty }$	${\mathrm{IL}}_{12}^{\infty }$	${\mathrm{IL}}_{10}^{\infty }$	$E^{\infty }$	$I_{\gamma }^{\infty }$	${\mathrm{IL}}_{6}^{\infty }$	
1	$1.15\times 10^{4}$	$9.01\times 10^{4}$	5.61	6.42	3.17	8.86	3.21	3.42	
2	$1.02\times 10^{4}$	$9.14\times 10^{4}$	3.84	2.84	1.36	4.03	1.19	1.28	
3	$8.11\times 10^{3}$	$8.80\times 10^{4}$	4.49	4.02	2.13	7.68	1.97	2.14	
4	$1.54\times 10^{4}$	$9.22\times 10^{4}$	7.54	1.04	5.08	1.37	5.72	5.80	
5	$1.53\times 10^{4}$	$7.67\times 10^{4}$	7.70	1.02	5.05	1.50	5.17	6.01	

**Table 4:** **Three important parameter values.** Values of the parameters $\delta_{CT_{c}}$, $\delta_{CI_{\gamma }}$, and $\delta_{C}$ without scaling and after scaling the assumption (24) by factors 0.2 and 5. These are the most effected parameters and correspond to the dynamics in Figure 8A.

Clusters	Without Scaling	Scale = 0.2	Scale = 5
Cluster 1	$\delta_{CT_{c}}=0.00440$ $\delta_{CI_{\gamma }}=0.00274$ $\delta_{C}=0.04492$	$\delta_{CT_{c}}=0.00895$ $\delta_{CI_{\gamma }}=0.00111$ $\delta_{C}=0.01827$	$\delta_{CT_{c}}=0.00124$ $\delta_{CI_{\gamma }}=0.00390$ $\delta_{C}=0.06341$
Cluster 2	$\delta_{CT_{c}}=0.00528$ $\delta_{CI_{\gamma }}=0.00074$ $\delta_{C}=0.03273$	$\delta_{CT_{c}}=0.00953$ $\delta_{CI_{\gamma }}=0.00027$ $\delta_{C}=0.01182$	$\delta_{CT_{c}}=0.00163$ $\delta_{CI_{\gamma }}=0.00115$ $\delta_{C}=0.05063$
Cluster 3	$\delta_{CT_{c}}=0.00494$ $\delta_{CI_{\gamma }}=0.00165$ $\delta_{C}=0.04421$	$\delta_{CT_{c}}=0.01027$ $\delta_{CI_{\gamma }}=0.00069$ $\delta_{C}=0.01839$	$\delta_{CT_{c}}=0.00137$ $\delta_{CI_{\gamma }}=0.00230$ $\delta_{C}=0.06150$
Cluster 4	$\delta_{CT_{c}}=0.00805$ $\delta_{CI_{\gamma }}=0.00283$ $\delta_{C}=0.02606$	$\delta_{CT_{c}}=0.01183$ $\delta_{CI_{\gamma }}=0.00083$ $\delta_{C}=0.00766$	$\delta_{CT_{c}}=0.00310$ $\delta_{CI_{\gamma }}=0.00546$ $\delta_{C}=0.05018$
Cluster 5	$\delta_{CT_{c}}=0.00676$ $\delta_{CI_{\gamma }}=0.00298$ $\delta_{C}=0.03037$	$\delta_{CT_{c}}=0.01068$ $\delta_{CI_{\gamma }}=0.00094$ $\delta_{C}=0.00960$	$\delta_{CT_{c}}=0.00238$ $\delta_{CI_{\gamma }}=0.00526$ $\delta_{C}=0.05355$

**Table 5:** **Three important parameter values.** Values of the parameters $\delta_{CT_{c}}$, $\delta_{CI_{\gamma }}$, and $\delta_{C}$ without scaling and after scaling the assumption (25) by factors 0.2 and 5. These are the most effected parameters and correspond to the dynamics in Figure 8B.

Cluster	Without Scaling	Scale = 0.2	Scale = 5
Cluster 1	$\delta_{CT_{c}}=0.00440$ $\delta_{CI_{\gamma }}=0.00274$ $\delta_{C}=0.04492$	$\delta_{CT_{c}}=0.00168$ $\delta_{CI_{\gamma }}=0.00104$ $\delta_{C}=0.08556$	$\delta_{CT_{c}}=0.00652$ $\delta_{CI_{\gamma }}=0.00406$ $\delta_{C}=0.013308$
Cluster 2	$\delta_{CT_{c}}=0.00528$ $\delta_{CI_{\gamma }}=0.00074$ $\delta_{C}=0.03273$	$\delta_{CT_{c}}=0.00161$ $\delta_{CI_{\gamma }}=0.00023$ $\delta_{C}=0.05002$	$\delta_{CT_{c}}=0.00967$ $\delta_{CI_{\gamma }}=0.00136$ $\delta_{C}=0.01199$
Cluster 3	$\delta_{CT_{c}}=0.00494$ $\delta_{CI_{\gamma }}=0.00165$ $\delta_{C}=0.04421$	$\delta_{CT_{c}}=0.00155$ $\delta_{CI_{\gamma }}=0.00052$ $\delta_{C}=0.06927$	$\delta_{CT_{c}}=0.00879$ $\delta_{CI_{\gamma }}=0.00294$ $\delta_{C}=0.01574$
Cluster 4	$\delta_{CT_{c}}=0.00805$ $\delta_{CI_{\gamma }}=0.00283$ $\delta_{C}=0.02606$	$\delta_{CT_{c}}=0.00467$ $\delta_{CI_{\gamma }}=0.00164$ $\delta_{C}=0.07563$	$\delta_{CT_{c}}=0.00941$ $\delta_{CI_{\gamma }}=0.00331$ $\delta_{C}=0.00609$
Cluster 5	$\delta_{CT_{c}}=0.00676$ $\delta_{CI_{\gamma }}=0.00298$ $\delta_{C}=0.03037$	$\delta_{CT_{c}}=0.00358$ $\delta_{CI_{\gamma }}=0.00158$ $\delta_{C}=0.08036$	$\delta_{CT_{c}}=0.00823$ $\delta_{CI_{\gamma }}=0.00363$ $\delta_{C}=0.00739$

**Figure A1:**
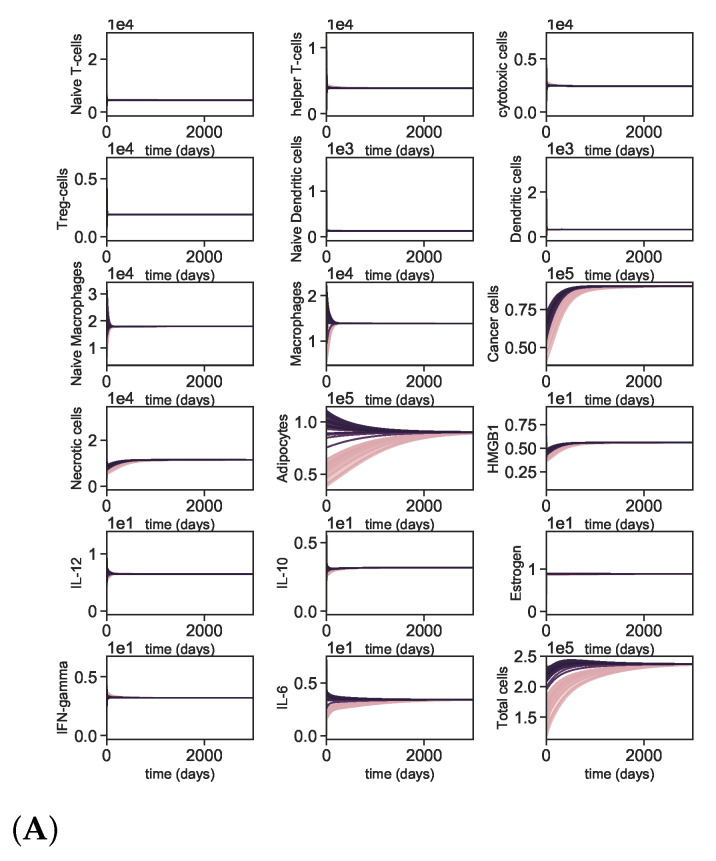

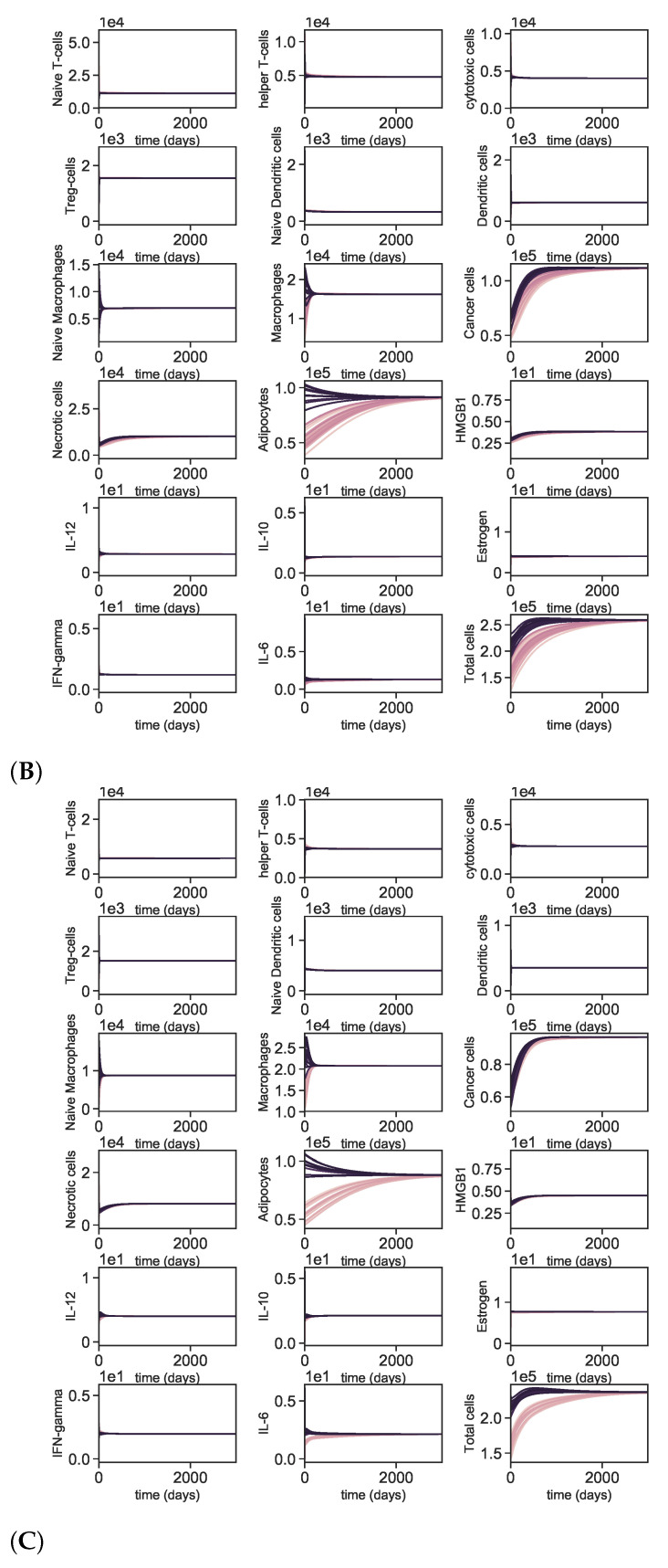

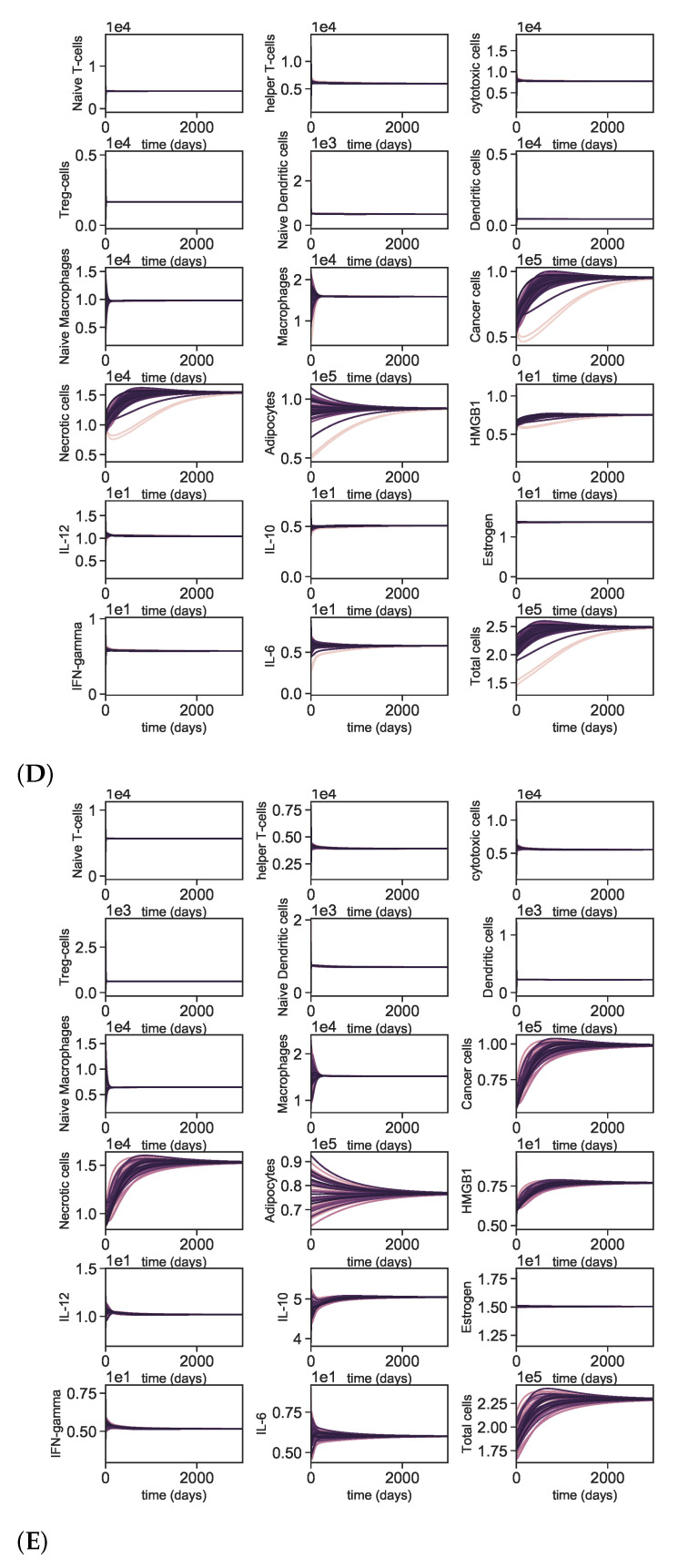
**The dynamics with varying initial conditions**. Subfigures (**A**–**E**) show the dynamics of cells and cytokines with initial conditions from different patients in clusters 1, 2, 3, 4, and 5, respectively.

**Figure A2:**
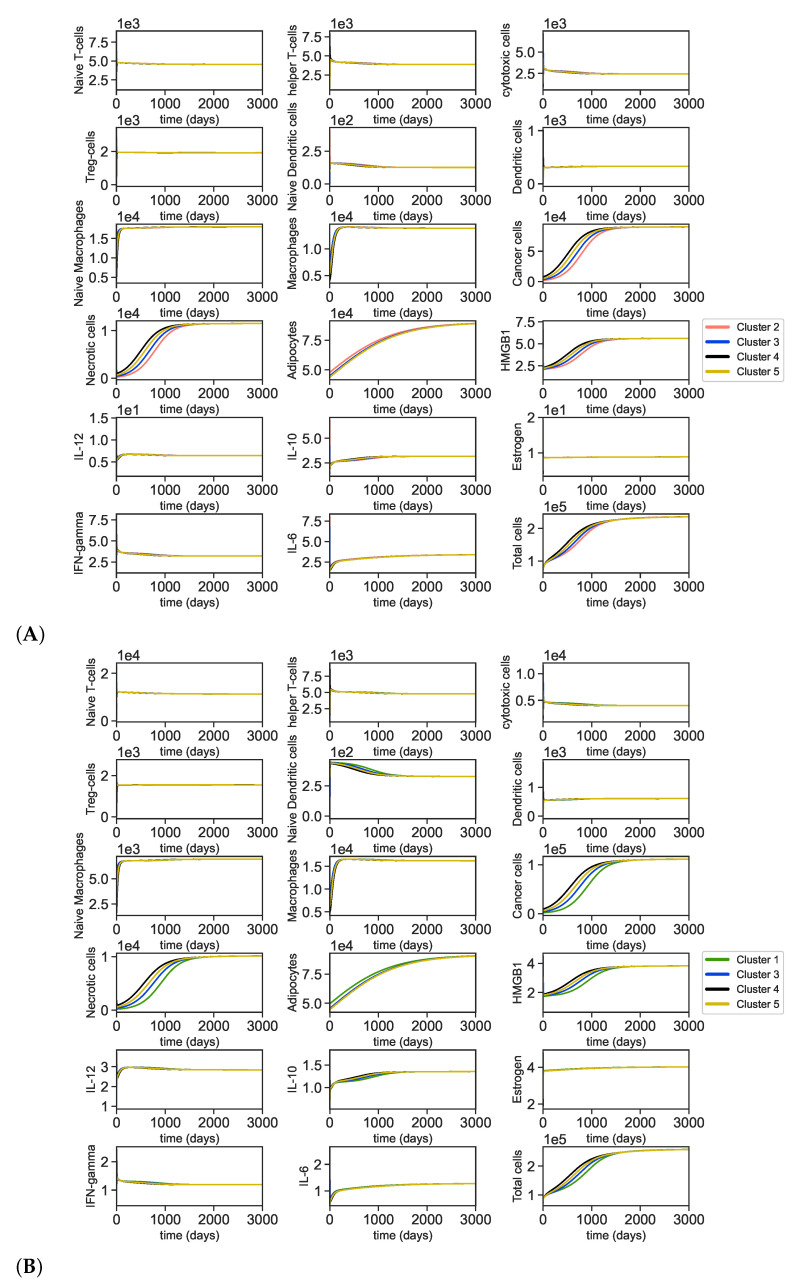

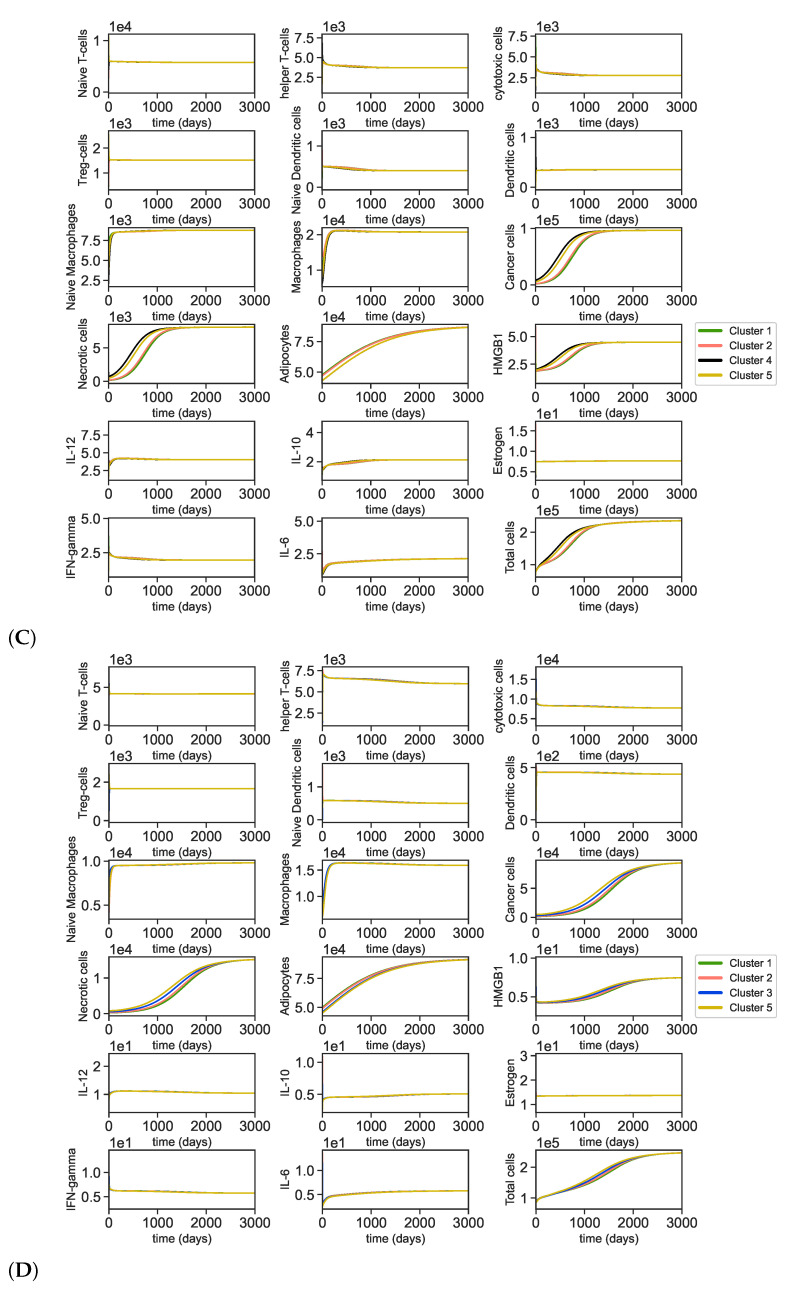

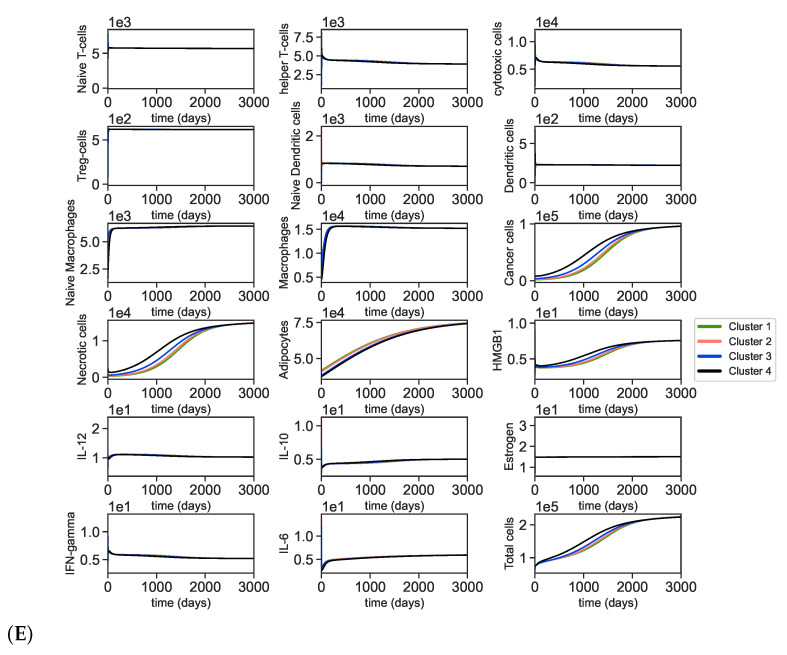
**Dynamics with cross-cluster initial conditions:** (**A**) parameters from cluster 1 and initial conditions from clusters 2, 3, 4, and 5; (**B**) parameters from cluster 2 and initial conditions from clusters 1, 3, 4, and 5; (**C**) parameters from cluster 3 and initial conditions from clusters 1, 2, 4, and 5; (**D**) parameters from cluster 4 and initial conditions from clusters 1, 2, 3, and 5; and (**E**) parameters from cluster 5 and initial conditions from clusters 1, 2, 3, and 4.

## Data Availability

The TCGA datasets of BRCA project are available at https://portal.gdc.cancer.gov and https://xenabrowser.net/datapages/, accessed on 1 June 2021. The METABRIC dataset is available at https://www.cbioportal.org/datasets, accessed on 1 June 2021. Codes are available at our GitHub page https://github.com/ShahriyariLab/Mathematical-Model-of-Breast-Tumors-Progression-Based-on-Their-Immune-Infiltration, accessed on 1 June 2021.

## Abbreviations

The following abbreviations are used in this manuscript:

TCGA	The Cancer Genome Atlas
METABRIC	Molecular Taxonomy of Breast Cancer International Consortium
HMGB1	High mobility group box-1
LumA	Luminal A
LumB	Luminal B
TNBC	Triple negative breast cancer
IFN-γ	Interferon gamma
HER2	Human epidermal growth factor 2
ER	Estrogen receptor
DAMP	Damage-associated molecular pattern
UCSC	University of Santa Cruz

## Appendix A. Derivation of Sample Parameters

### Appendix A.1. Steady State and Additional Assumptions

Since we were modeling the evolutions of tumors without any interventions, we assumed small tumors grow to large ones at a steady state. As there were both small and large tumors in each cluster, the averages of the smallest tumors and the largest tumors in each cluster, respectively, gave us the initial conditions and the steady state values for the cluster; see Table 2 and Table 3. However, we carried out our parameter estimation based on just the steady state values and independent of the initial conditions. This enabled us to validate the importance of our parameter values based on the data of every single patient in the same cluster and across clusters; see Appendices Appendix A.4 and Appendix A.5.

If we assume specific values of steady state for each variable, we obtain the following sample parameter set:

$$\begin{array}{c} T_{N}^{\infty },\;T_{c}^{\infty },\;T_{h}^{\infty },\;T_{r}^{\infty },\;D_{N}^{\infty },\;D^{\infty },\;M^{\infty },\;M_{N}^{\infty },\;A^{\infty },\;C^{\infty },\;N^{\infty },\;H^{\infty },\;E^{\infty },\;IL_{6}^{\infty },\;IL_{10}^{\infty },\;IL_{12}^{\infty }\;\mathrm{and}\;I_{\gamma }^{\infty }. \end{array}$$

Under these assumptions, the system of Equations (1)–(17) provided us with 17 constraints on parameters. In order to solve this system, we needed to make additional assumptions about a selection of parameters. This ensured that the number of independent equations equaled the number of unknown parameters and that we could uniquely determine the values. We assumed that the carrying capacities $A_{0}$, $C_{0}$, and $M_{0}$ were proportional to the maximum values of their corresponding variables in each cluster. We also took the necrosis coefficient to be $\alpha_{NC}=0.5$. We also utilized the available research [71,121,122,123,124] to adopt the natural death/decay/degradation rates. Considering a cell or cytokine *X*, we estimated the death rate $\delta_{X}=\ln2/t_{1/2}^{X}$ using half-life measurements $t_{1/2}^{X}$.

More specifically, we found that naive T-cells’ half-life ranges between 1 and 8 years [71]. Taking it to be two years, we derived a death rate of $9.49\times 10^{-4}$. Next, we found that the half-lives of cytotoxic T-cells and helper T-cells are 41 h and 3 days, respectively, resulting in $\delta_{T_{h}}=\delta_{T_{r}}=2.31$ and $\delta_{T_{c}}=0.406$ [71]. The half-life of mature DCs is about 2 to 3 days, giving us an average of 2.5 days. Using this value for $t_{1/2}^{X}$, we obtained $\delta_{D}=0.277$. Intestinal macrophages have a half life ranging from 4 to 6 weeks. Considering the half life of five weeks, we calculated $\delta_{M}=0.0198$. According to [125], we found that the half life of adipocytes is 10 years, resulting in $\delta_{A}=2.8\times 10^{-3}$. For HMGB1, there are varying results in [121]. We considered a half-life of 17 min and used this to find $\delta_{H}=18$. For the degradation rate of estrogen, we considered a 3 to 5 h half-life [122]. Using the average of 4 h, we calculated $\delta_{E}=4.16$. For IL-6, we assumed the half-life to be 15.5 h. Thus, we obtained $\delta_{IL_{6}}=1.07$ [71]. We took the half-lives of IL-10 and IL-12 to be 3.6 h [71] and 7.8 min [123], respectively. This resulted in $\delta_{IL_{10}}=4.62$ and $\delta_{IL_{12}}=128$. Lastly, for IFN-γ, the estimated half-life was about 30 min, resulting in the rate $\delta_{\gamma }=33.3$. More concisely, we used the following the degradation rates in our computation:

$$\begin{array}{cccccccc} \delta_{T_{N}} & =9.49\times 10^{-4} & \delta_{T_{c}} & =0.406 & \delta_{T_{h}} & =0.231 & \delta_{T_{r}} & =0.231\\ \delta_{D} & =0.277 & \delta_{M} & =0.0198 & \delta_{A} & =2.8\times 10^{-3} & \delta_{H} & =18\\ \delta_{E} & =4.16 & \delta_{IL_{6}} & =1.07 & \delta_{IL_{10}} & =4.62 & \delta_{IL_{12}} & =128\\ \delta_{I_{\gamma }} & =33.3 \end{array}$$

Though this decreased the number of unknowns, there still existed a substantial number of parameters without values. Therefore, we next imposed heuristic assumptions based on activation, production, and inhibition rates. Let us look at those in detail.

We began by considering that the mean rate of doubling time of a breast cancer tumor is 193±141 [126]. This gives an interval of 52 days to 334 days. Using this fact, we assumed that a faster doubling rate results from both innate cancer proliferation and proliferation caused by cytokine IL-6 and adipocytes. In the case of the faster double rate, we assumed that death is only innate. This results in

(A1) $$\frac{\ln2}{52}\approx 1.3330\times 10^{-2}=\;\left(\lambda_{C}+\lambda_{CIL_{6}}\left[IL_{6}^{\mathrm{mean}}\right]+\lambda_{CA}\left[A^{\mathrm{mean}}\right]\right)-\delta_{C}.$$

This contrasts with our slower doubling rate assumptions. We assumed only innate cancer proliferation. In this case, death rates included the effects of anti-cancer agents cytotoxic T-cells and IFN-γ, giving us

(A2) $$\frac{\ln2}{334}\approx 2.0753\times 10^{3}=\lambda_{C}-\left(\delta_{CT_{c}}\left[T_{c}^{\mathrm{mean}}\right]+\delta_{CI_{\gamma }}\left[I_{\gamma }^{\mathrm{mean}}\right]+\delta_{C}\right).$$

We used the following mean values:

**Table A1:** **Mean values of the variables used.** The mean values were calculated from the combination of TCGA and METABRIC datasets for all of the clusters.

${\mathrm{IL}}_{6}^{\mathrm{mean}}$	$A^{\mathrm{mean}}$	$T_{c}^{\mathrm{mean}}$	$I_{\gamma }^{\mathrm{mean}}$
3.906	$7.112\times 10^{4}$	$4.129\times 10^{3}$	3.675

In these assumptions, we took $IL_{6}^{\mathrm{mean}}$, $A^{\mathrm{mean}}$, $T_{c}^{\mathrm{mean}}$, and $I_{\gamma }^{\mathrm{mean}}$ to be the average values of the corresponding variable across all patients. In our further assumptions, we used the maximum observable quantities for all the variables across all patients denoted by:

$$\begin{array}{cc} \; & T_{N}^{\max},\;T_{c}^{\max},\;T_{h}^{\max},\;T_{r}^{\max},\;D_{N}^{\max},\;D^{\max},\;M_{N}^{\max},\;M^{\max},\;A^{\max},\;C^{\max},\;N^{\max},\;H^{\max},\;E^{\max},\;\\ \; & IL_{6}^{\max},\;IL_{10}^{\max},\;IL_{12}^{\max},\;\mathrm{and}\;I_{\gamma }^{\max}. \end{array}$$

The following maximum variables were calculated from the datasets:

**Table A2:** **Maximum values of the variables.** The maximum values were calculated from the combination of TCGA and METABRIC datasets for all of the clusters.

$T_{N}^{\max}$	$T_{c}^{\max}$	$T_{h}^{\max}$	$T_{r}^{\max}$	$D_{N}^{\max}$	$D^{\max}$
$4.270\times 10^{4}$	$1.599\times 10^{4}$	$2.495\times 10^{4}$	$1.174\times 10^{4}$	$6.507\times 10^{3}$	$8.274\times 10^{3}$
$M_{N}^{\max}$	$M^{\max}$	$N^{\max}$	$N^{\max}$	$A^{\max}$	$H^{\max}$
$4.577\times 10^{4}$	$5.247\times 10^{4}$	$4.258\times 10^{5}$	$1.019\times 10^{5}$	$2.612\times 10^{5}$	$1.160\times 10^{1}$
$IL_{12}^{\max}$	$IL_{10}^{\max}$	$E^{\max}$	$I_{\gamma }^{\max}$	$IL_{6}^{\max}$	
$2.516\times 10^{1}$	5.962	$1.868\times 10^{1}$	8.768	$1.073\times 10^{1}$	

Assuming helper T-cells are primarily activated by dendritic cells, we can write

(A3) $$\lambda_{T_{h}D}\left[D^{\max}\right]=200\lambda_{T_{h}H}\left[H^{\max}\right]=200\lambda_{T_{h}IL_{12}}\left[IL_{12}^{\max}\right]=200\lambda_{T_{h}E}\left[E^{\max}\right].$$

We took the inhibition of helper T-cells to be mostly accomplished by cytokine IL-10 and regulatory T-cells. In particular, we claim that these inhibitors are 20 times more effective than natural degradation.

(A4) $$\delta_{T_{h}T_{r}}\left[T_{r}^{\max}\right]=\delta_{T_{h}IL_{10}}\left[IL_{10}^{\max}\right]=20\delta_{T_{h}}.$$

Next, we made the following assumptions about cytotoxic T-cells. The activation of cytotoxic T-cells by estrogen is much stronger than activation by IL-12 and dendritic cells. We declare estrogen to be 200 times as effective as IL-12, and we declare dendrites to be 100 times as effective as estrogen.

(A5) $$\lambda_{T_{c}E}\left[E^{\max}\right]=100\lambda_{T_{c}D}\left[D^{\max}\right]=200\lambda_{T_{c}IL_{12}}\left[IL_{12}^{\max}\right].$$

We also state that IL-10 and regulatory T-cells are 20 times more effective than natural degradation of cytotoxic T-cells.

(A6) $$\delta_{T_{c}T_{r}}\left[T_{r}^{\max}\right]=\delta_{T_{c}IL_{10}}\left[IL_{10}^{\max}\right]=20\delta_{T_{c}}.$$

For regulatory T-cells, we assumed that their activation by dendritic cells is four times stronger than their activation by estrogen. This is written as

(A7) $$\lambda_{T_{r}D}\left[D^{\max}\right]=4\lambda_{T_{r}E}\left[E^{\max}\right].$$

We also claim that the activation of dendritic cells by HMGB1 and estrogen is twice as effective as by cancer cells. This gives us the following equality:

(A8) $$2\lambda_{DC}\left[C^{\max}\right]=\lambda_{DH}\left[H^{\max}\right]=\lambda_{DE}\left[E^{\max}\right].$$

Next, we declare the inhibition of dendritic cells by cancer cells to be equivalent to the natural degradation rate. In other words,

(A9) $$\delta_{DC}\left[C^{\max}\right]=\delta_{D}.$$

Further, we assume that helper T-cells are the primary activator for macrophages. We express this assumption as

(A10) $$10\lambda_{MIL_{10}}\left[IL_{10}^{\max}\right]=10\lambda_{MI_{\gamma }}\left[I_{\gamma }^{\max}\right]=10\lambda_{MIL_{12}}\left[IL_{12}^{\max}\right]=\lambda_{MT_{h}}\left[T_{h}^{\max}\right]=10\lambda_{ME}\left[E^{\max}\right].$$

For HMGB1, we declare the following equality to be true.

(A11) $$\lambda_{HD}\left[D^{\max}\right]=\lambda_{HN}\left[N^{\max}\right]=10\lambda_{HM}\left[M^{\max}\right]=\lambda_{HT_{c}}\left[T_{c}^{\max}\right]=\lambda_{HC}\left[C^{\max}\right].$$

Our next assumption sets the production of IL-12 to be equal among macrophages, dendritic cells, helper T-cells, and cytotoxic T-cells:

(A12) $$\lambda_{IL_{12}M}\left[M^{\max}\right]=\lambda_{IL_{12}D}\left[D^{\max}\right]=\lambda_{IL_{12}T_{h}}\left[T_{h}^{\max}\right]=\lambda_{IL_{12}T_{c}}\left[T_{c}^{\max}\right].$$

For the production of IL-10, we let macrophages, dendritic cells, regulatory T-cells, helper T-cells, cytotoxic T-cells, and cancer cells work at equal rates:

(A13) $$\begin{array}{cc} \lambda_{IL_{10}M}\left[M^{\max}\right] & =\lambda_{IL_{10}D}\left[D^{\max}\right]=\lambda_{IL_{10}T_{r}}\left[T_{r}^{\max}\right]=\lambda_{IL_{10}T_{h}}\left[T_{h}^{\max}\right]\\ \; & =\lambda_{IL_{10}T_{c}}\left[T_{c}^{\max}\right]=\lambda_{IL_{10}C}\left[C^{\max}\right]. \end{array}$$

Next, we assumed that IFN-γ is mostly produced by cytotoxic T-cells. This gave us the following equality:

(A14) $$\lambda_{I_{\gamma }T_{c}}\left[T_{c}^{\max}\right]=5\lambda_{I_{\gamma }T_{h}}\left[T_{h}^{\max}\right]=5\lambda_{I_{\gamma }D}\left[D^{\max}\right].$$

We also declare IL-6 to be equally produced by adipocytes, macrophages, and dendritic cells. Thus, we obtained the following:

(A15) $$\lambda_{IL_{6}A}\left[A^{\max}\right]=\lambda_{IL_{6}M}\left[M^{\max}\right]=\lambda_{IL_{6}D}\left[D^{\max}\right].$$

Furthermore, we scaled the carrying capacities for cancer cells, adipocytes, and estrogen by 2, 2, and 1.5, respectively.

(A16) $$\begin{array}{c} C_{0}=2\times \left[C^{\max}\right],\;A_{0}=2\times \left[A^{\max}\right],\;\mathrm{and}\;E_{0}=1.5\times \left[E^{\max}\right]. \end{array}$$

We took the necrosis factor $\alpha_{NC}$ to be 0.5 for all clusters.

(A17) $$\alpha_{NC}=0.5.$$

The proliferation rate of cancer cells by $IL_{6}$ was assumed to be twice that by adipocytes:

(A18) $$2\lambda_{CA}\left[A^{\max}\right]=\lambda_{CIL_{6}}\left[IL_{6}^{\max}\right].$$

We declare the general secretion of estrogen to be twenty times the production of estrogen by adipocytes:

(A19) $$20\lambda_{EA}\left[A^{\max}\right]=\lambda_{E}.$$

We took the depreciation of naive dendritic cells to be equivalent to that of dendritic cells. Likewise, we took the depreciation of naive macrphage cells to be equivalent to that of macrophages.

(A20) $$\begin{array}{c} \delta_{D_{N}}=\delta_{D} \end{array}$$

(A21) $$\begin{array}{c} \delta_{M_{N}}=\delta_{M}. \end{array}$$

Finally, we assumed that the inhibition of cancer cells by IFN-γ is less effective than inhibition by cytotoxic cells and other causes:

(A22) $$\delta_{C}=\delta_{CT_{c}}\left[T_{c}^{\max}\right]=6\delta_{CI_{\gamma }}\left[I_{\gamma }^{\max}\right]$$

Altogether, these assumptions adequately allowed us to derive a sample parameter set.

### Appendix A.2. Non-Dimensionalization

For a more stable numerical simulation, parameter estimation, and sensitivity analysis, we non-dimensionalized our parameters and variables with respect to each variable’s steady state value. Therefore, a non-dimensional variable $\left[\bar{X}\right]$, non-dimensionalized as $\left[X\right]/\left[X^{\infty }\right]$, will take the value 1 at its steady state. Since the dimensional time scale does not introduce any inconveniences, we chose not to non-dimensionalize with respect to the time. As a result we got the following system:

(A23) $$\begin{array}{cc} \frac{d[\bar{T_{h}}]}{dt} & =\left({\bar{\lambda }}_{T_{h}H}\left[\bar{H}\right]+{\bar{\lambda }}_{T_{h}D}\left[\bar{D}\right]+{\bar{\lambda }}_{T_{h}IL_{12}}\left[\bar{IL_{12}}\right]+{\bar{\lambda }}_{T_{h}E}\left[\bar{E}\right]\right)\left[\bar{T_{N}}\right]\\ \; & -\left({\bar{\delta }}_{T_{h}T_{r}}\left[\bar{T_{r}}\right]+{\bar{\delta }}_{T_{h}IL_{10}}\left[\bar{IL_{10}}\right]+\delta_{T_{h}}\right)\left[\bar{T_{h}}\right], \end{array}$$

(A24) $$\begin{array}{cc} \frac{d[\bar{T_{c}}]}{dt} & =\left({\bar{\lambda }}_{T_{c}E}\left[\bar{E}\right]+{\bar{\lambda }}_{T_{c}D}\left[\bar{D}\right]+{\bar{\lambda }}_{T_{c}IL_{12}}\left[\bar{IL_{12}}\right]\right)\left[\bar{T_{N}}\right]\\ \; & -\left({\bar{\delta }}_{T_{c}T_{r}}\left[\bar{T_{r}}\right]+{\bar{\delta }}_{T_{c}IL_{10}}\left[\bar{IL_{10}}\right]+\delta_{T_{c}}\right)\left[\bar{T_{c}}\right], \end{array}$$

(A25) $$\frac{d[\bar{T_{r}}]}{dt}=\left({\bar{\lambda }}_{T_{r}D}\left[\bar{D}\right]+{\bar{\lambda }}_{T_{r}E}\left[\bar{E}\right]\right)\left[\bar{T_{N}}\right]-\delta_{T_{r}}\left[\bar{T_{r}}\right],$$

(A26) $$\begin{array}{cc} \frac{d[\bar{T_{N}}]}{dt} & ={\bar{A}}_{T_{N}}-\left({\bar{\lambda }}_{T_{h}H}\left[\bar{H}\right]+{\bar{\lambda }}_{T_{h}D}\left[\bar{D}\right]+{\bar{\lambda }}_{T_{h}IL_{12}}\left[\bar{IL_{12}}\right]+{\bar{\lambda }}_{T_{h}E}\left[\bar{E}\right]\right)\left[\bar{T_{N}}\right]\\ \; & -\left({\bar{\lambda }}_{T_{c}E}\left[\bar{E}\right]+{\bar{\lambda }}_{T_{c}D}\left[\bar{D}\right]+{\bar{\lambda }}_{T_{c}IL_{12}}\left[\bar{IL_{12}}\right]\right)\left[\bar{T_{N}}\right]\\ \; & -\left({\bar{\lambda }}_{T_{r}D}\left[\bar{D}\right]+{\bar{\lambda }}_{T_{r}E}\left[\bar{E}\right]+\delta_{T_{N}}\right)\left[\bar{T_{N}}\right], \end{array}$$

(A27) $$\begin{array}{cc} \frac{d[\bar{D_{N}}]}{dt} & ={\bar{A}}_{D_{N}}-\left({\bar{\lambda }}_{DC}\left[\bar{C}\right]+{\bar{\lambda }}_{DH}\left[\bar{H}\right]+{\bar{\lambda }}_{DE}\left[\bar{E}\right]\right)\left[\bar{D_{N}}\right]-\delta_{D_{N}}\left[\bar{D_{N}}\right], \end{array}$$

(A28) $$\begin{array}{cc} \frac{d[\bar{D}]}{dt} & =\left({\bar{\lambda }}_{DC}\left[\bar{C}\right]+{\bar{\lambda }}_{DH}\left[\bar{H}\right]+{\bar{\lambda }}_{DE}\left[\bar{E}\right]\right)\left[\bar{D_{N}}\right]-\left({\bar{\delta }}_{DC}\left[\bar{C}\right]+\delta_{D}\right)\left[\bar{D}\right], \end{array}$$

(A29) $$\begin{array}{cc} \frac{d[\bar{M_{N}}]}{dt} & ={\bar{A}}_{M}-({\bar{\lambda }}_{MIL_{10}}\left[\bar{IL_{10}}\right]+{\bar{\lambda }}_{MI_{\gamma }}\left[\bar{I_{\gamma }}\right]+{\bar{\lambda }}_{MIL_{12}}\left[\bar{IL_{12}}\right]+{\bar{\lambda }}_{MT_{h}}\left[\bar{T_{h}}\right]\\ \; & +{\bar{\lambda }}_{ME}\left[\bar{E}\right]+\delta_{M_{N}})\left[\bar{M_{N}}\right], \end{array}$$

(A30) $$\begin{array}{cc} \frac{d[\bar{M}]}{dt} & =\left({\bar{\lambda }}_{MIL_{10}}\left[\bar{IL_{10}}\right]+{\bar{\lambda }}_{MI_{\gamma }}\left[\bar{I_{\gamma }}\right]+{\bar{\lambda }}_{MIL_{12}}\left[\bar{IL_{12}}\right]+{\bar{\lambda }}_{MT_{h}}\left[\bar{T_{h}}\right]+{\bar{\lambda }}_{ME}\left[\bar{E}\right]\right)\left[\bar{M_{N}}\right]\\ \; & -\delta_{M}\left[\bar{M}\right], \end{array}$$

(A31) $$\begin{array}{cc} \frac{d[\bar{C}]}{dt} & =\left(\lambda_{C}+{\bar{\lambda }}_{CIL_{6}}\left[\bar{IL_{6}}\right]+{\bar{\lambda }}_{CA}\left[\bar{A}\right]\right)\left(1-\frac{\left[\bar{C}\right]}{\bar{C_{0}}}\right)\left[\bar{C}\right]\\ \; & -\left({\bar{\delta }}_{CT_{c}}\left[\bar{T_{c}}\right]+{\bar{\delta }}_{CI_{\gamma }}\left[\bar{I_{\gamma }}\right]+\delta_{C}\right)\left[\bar{C}\right], \end{array}$$

(A32) $$\begin{array}{cc} \frac{d[\bar{A}]}{dt} & =\lambda_{A}\left[\bar{A}\right]\left(1-\frac{\left[\bar{A}\right]}{\bar{A_{0}}}\right)-\delta_{A}\left[\bar{A}\right], \end{array}$$

(A33) $$\begin{array}{cc} \frac{d[\bar{N}]}{dt} & ={\bar{\alpha }}_{NC}\left({\bar{\delta }}_{CI_{\gamma }}\left[\bar{I_{\gamma }}\right]+{\bar{\delta }}_{CT_{c}}\left[\bar{T_{c}}\right]+\delta_{C}\right)\left[\bar{C}\right]-\delta_{N}\left[\bar{N}\right], \end{array}$$

(A34) $$\begin{array}{cc} \frac{d[\bar{H}]}{dt} & ={\bar{\lambda }}_{HD}\left[\bar{D}\right]+{\bar{\lambda }}_{HN}\left[\bar{N}\right]+{\bar{\lambda }}_{HM}\left[\bar{M}\right]+{\bar{\lambda }}_{HT_{c}}\left[\bar{T_{c}}\right]+{\bar{\lambda }}_{HC}\left[\bar{C}\right]-\delta_{H}\left[\bar{H}\right], \end{array}$$

(A35) $$\begin{array}{cc} \frac{d[\bar{IL_{12}}]}{dt} & ={\bar{\lambda }}_{IL_{12}M}\left[\bar{M}\right]+{\bar{\lambda }}_{IL_{12}D}\left[\bar{D}\right]+{\bar{\lambda }}_{IL_{12}T_{h}}\left[\bar{T_{h}}\right]+{\bar{\lambda }}_{IL_{12}T_{c}}\left[\bar{T_{c}}\right]-\delta_{IL_{12}}\left[\bar{IL_{12}}\right], \end{array}$$

(A36) $$\begin{array}{cc} \frac{d[\bar{IL_{10}}]}{dt} & ={\bar{\lambda }}_{IL_{10}M}\left[\bar{M}\right]+{\bar{\lambda }}_{IL_{10}D}\left[\bar{D}\right]+{\bar{\lambda }}_{IL_{10}T_{r}}\left[\bar{T_{r}}\right]+{\bar{\lambda }}_{IL_{10}T_{h}}\left[\bar{T_{h}}\right]+{\bar{\lambda }}_{IL_{10}T_{c}}\left[\bar{T_{c}}\right]+{\bar{\lambda }}_{IL_{10}C}\left[\bar{C}\right]\\ \; & -\delta_{IL_{10}}\left[\bar{IL_{10}}\right], \end{array}$$

(A37) $$\begin{array}{cc} \frac{d[\bar{E}]}{dt} & ={\bar{\lambda }}_{EA}\left[\bar{A}\right]+\lambda_{E}\left[\bar{E}\right]\left(1-\frac{\left[\bar{E}\right]}{\bar{E_{0}}}\right)-\delta_{E}\left[\bar{E}\right], \end{array}$$

(A38) $$\begin{array}{cc} \frac{d[\bar{I_{\gamma }}]}{dt} & ={\bar{\lambda }}_{I_{\gamma }T_{c}}\left[\bar{T_{c}}\right]+{\bar{\lambda }}_{I_{\gamma }T_{h}}\left[\bar{T_{h}}\right]+{\bar{\lambda }}_{I_{\gamma }D}\left[\bar{E}\right]\left[\bar{D}\right]-\delta_{I_{\gamma }}\left[\bar{I_{\gamma }}\right], \end{array}$$

(A39) $$\begin{array}{cc} \frac{d[\bar{IL_{6}}]}{dt} & ={\bar{\lambda }}_{IL_{6}A}\left[\bar{A}\right]+{\bar{\lambda }}_{IL_{6}M}\left[\bar{M}\right]+{\bar{\lambda }}_{IL_{6}D}\left[\bar{D}\right]-\delta_{IL_{6}}\left[\bar{IL_{6}}\right]. \end{array}$$

Since we did not nondimensionalize with respect to time, the production rates $\lambda_{C},\lambda_{A}$ and $\lambda_{E}$ and the decay rates $\delta_{T_{h}}$, $\delta_{T_{c}}$, $\delta_{T_{r}}$, $\delta_{T_{N}}$, $\delta_{D_{N}}$, $\delta_{D}$, $\delta_{M_{N}}$, $\delta_{M}$, $\delta_{C}$, $\delta_{A}$, $\delta_{N}$, $\delta_{H}$, $\delta_{IL_{12}}$, $\delta_{IL_{10}}$, $\delta_{E}$, $\delta_{I_{\gamma }}$, $\delta_{T_{h}}$, and $\delta_{IL_{6}}$ were left unchanged. To non-dimensionalize the rest of the parameters, we used the following rules:

(A40) $$\begin{array}{cccccc} {\bar{A}}_{T_{N}} & =\frac{A_{T_{N}}}{\left[T_{N}^{\infty }\right]}, & {\bar{A}}_{D_{N}} & =\frac{A_{D_{N}}}{\left[D_{N}^{\infty }\right]}, & {\bar{A}}_{M} & =\frac{A_{M}}{\left[M^{\infty }\right]},\\ {\bar{\alpha }}_{NC} & =\alpha_{NC}\frac{\left[C^{\infty }\right]}{\left[N^{\infty }\right]},\; & {\bar{C}}_{0} & =\frac{C_{0}}{\left[C^{\infty }\right]}, & {\bar{A}}_{0} & =\frac{A_{0}}{\left[A^{\infty }\right]},\\ {\bar{E}}_{0} & =\frac{E_{0}}{\left[E^{\infty }\right]}. \end{array}$$

Furthermore, for the reaction parameters of the form ${\bar{\lambda }}_{XY}$ used in terms of the form ${\bar{\lambda }}_{XY}\left[Y\right]\left[Z\right]$, we used the general non-dimensinalization formula:

(A41) $$\begin{array}{cc} {\bar{\lambda }}_{XY} & =\frac{\lambda_{XY}\left[Y^{\infty }\right]\left[Z^{\infty }\right]}{\left[X^{\infty }\right]}. \end{array}$$

and finally, for inhibition rates of the form ${\bar{\delta }}_{XY}$ used in terms of the form ${\bar{\delta }}_{XY}\left[Y\right]\left[Z\right]$, we used the general non-dimensinalization formula:

(A42) $$\begin{array}{cc} {\bar{\delta }}_{XY} & =\delta_{XY}\left[Y^{\infty }\right]. \end{array}$$

Consequently, we got non-dimensionalized assumptions as follows:

(A43) $$\begin{array}{cc} & \frac{\ln2}{52}\approx 1.3330\times 10^{-2}=\left(\lambda_{C}+\lambda_{CIL_{6}}\frac{\left[IL_{6}^{\mathrm{mean}}\right]}{\left[IL_{6}^{\infty }\right]}+\lambda_{CA}\frac{\left[A^{\mathrm{mean}}\right]}{\left[A^{\infty }\right]}\right)-\delta_{C}, \end{array}$$

(A44) $$\begin{array}{cc} & \frac{\ln2}{334}\approx 2.0753\times 10^{3}=\lambda_{C}-\left(\delta_{CT_{c}}\frac{\left[T_{c}^{\mathrm{mean}}\right]}{\left[T_{c}^{\infty }\right]}+\delta_{CI_{\gamma }}\frac{\left[I_{\gamma }^{\mathrm{mean}}\right]}{\left[I_{\gamma }^{\infty }\right]}+\delta_{C}\right), \end{array}$$

(A45) $$\begin{array}{cc} & \lambda_{T_{h}D}\frac{\left[D^{\max}\right]}{\left[D^{\infty }\right]}=200\lambda_{T_{h}H}\frac{\left[H^{\max}\right]}{\left[H^{\infty }\right]}=200\lambda_{T_{h}IL_{12}}\frac{\left[IL_{12}^{\max}\right]}{\left[IL_{12}^{\infty }\right]}=200\lambda_{T_{h}E}\frac{\left[E^{\max}\right]}{\left[E^{\infty }\right]}, \end{array}$$

(A46) $$\begin{array}{cc} & \delta_{T_{h}T_{r}}\frac{\left[T_{r}^{\max}\right]}{\left[T_{r}^{\infty }\right]}=\delta_{T_{h}IL_{10}}\frac{\left[IL_{10}^{\max}\right]}{\left[IL_{10}^{\infty }\right]}=20\delta_{T_{h}},\;\lambda_{T_{r}D}\frac{\left[D^{\max}\right]}{\left[D^{\infty }\right]}=4\lambda_{T_{r}E}\frac{\left[E^{\max}\right]}{E^{\infty }}, \end{array}$$

(A47) $$\begin{array}{cc} & 2\lambda_{DC}\frac{\left[C^{\max}\right]}{\left[C^{\infty }\right]}=\lambda_{DH}\frac{\left[H^{\max}\right]}{\left[H^{\infty }\right]}=\lambda_{DE}\frac{\left[E^{\max}\right]}{\left[E^{\infty }\right]},\;\delta_{DC}\frac{\left[C^{\max}\right]}{\left[C^{\infty }\right]}=\delta_{D}, \end{array}$$

(A48) $$\begin{array}{cc} & 10\lambda_{MIL_{10}}\frac{\left[IL_{10}^{\max}\right]}{\left[IL_{10}^{\infty }\right]}=10\lambda_{MI_{\gamma }}\frac{\left[I_{\gamma }^{\max}\right]}{\left[I_{\gamma }^{\infty }\right]}=10\lambda_{MIL_{12}}\frac{\left[IL_{12}^{\max}\right]}{\left[IL_{12}^{\infty }\right]}=\lambda_{MT_{h}}\frac{\left[T_{h}^{\max}\right]}{\left[T_{h}^{\infty }\right]}=10\lambda_{ME}\frac{\left[E^{\max}\right]}{\left[E^{\infty }\right]}, \end{array}$$

(A49) $$\begin{array}{cc} & 50\delta_{CT_{c}}\frac{\left[T_{c}^{\max}\right]}{\left[T_{c}^{\infty }\right]}=\delta_{CI_{\gamma }}\frac{\left[I_{\gamma }^{\max}\right]}{\left[I_{\gamma }^{\infty }\right]}, \end{array}$$

(A50) $$\begin{array}{cc} & \lambda_{HD}\frac{\left[D^{\max}\right]}{\left[D^{\infty }\right]}=\lambda_{HN}\frac{\left[N^{\max}\right]}{\left[N^{\infty }\right]}=10\lambda_{HM}\frac{\left[M^{\max}\right]}{\left[M^{\infty }\right]}=\lambda_{HT_{c}}\frac{\left[T_{c}^{\max}\right]}{\left[T_{c}^{\infty }\right]}=\lambda_{HC}\frac{\left[C^{\max}\right]}{\left[C^{\infty }\right]}, \end{array}$$

(A51) $$\begin{array}{cc} & \lambda_{IL_{12}M}\frac{\left[M^{\max}\right]}{\left[M^{\infty }\right]}=\lambda_{IL_{12}D}\frac{\left[D^{\max}\right]}{\left[D^{\infty }\right]}=\lambda_{IL_{12}T_{h}}\frac{\left[T_{h}^{\max}\right]}{\left[T_{h}^{\infty }\right]}=\lambda_{IL_{12}T_{c}}\frac{\left[T_{c}^{\max}\right]}{\left[T_{c}^{\infty }\right]}, \end{array}$$

(A52) $$\begin{array}{cc} & \lambda_{IL_{10}M}\frac{\left[M^{\max}\right]}{\left[M^{\infty }\right]}=\lambda_{IL_{10}D}\frac{\left[D^{\max}\right]}{\left[D^{\infty }\right]}=\lambda_{IL_{10}T_{r}}\frac{\left[T_{r}^{\max}\right]}{\left[T_{r}^{\infty }\right]}=\lambda_{IL_{10}T_{h}}\frac{\left[T_{h}^{\max}\right]}{\left[T_{h}^{\infty }\right]}\\ & \;\;=\lambda_{IL_{10}T_{c}}\frac{\left[T_{c}^{\max}\right]}{\left[T_{c}^{\infty }\right]}=\lambda_{IL_{10}C}\frac{\left[C^{\max}\right]}{\left[C^{\infty }\right]}, \end{array}$$

(A53) $$\begin{array}{cc} & \lambda_{I_{\gamma }T_{c}}\frac{\left[T_{c}^{\max}\right]}{\left[T_{c}^{\infty }\right]}=5\lambda_{I_{\gamma }T_{h}}\frac{\left[T_{h}^{\max}\right]}{\left[T_{h}^{\infty }\right]}=5\lambda_{I_{\gamma }D}\frac{\left[D^{\max}\right]}{\left[D^{\infty }\right]}, \end{array}$$

(A54) $$\begin{array}{cc} & \lambda_{IL_{6}A}\frac{\left[A^{\max}\right]}{\left[A^{\infty }\right]}=\lambda_{IL_{6}M}\frac{\left[M^{\max}\right]}{\left[M^{\infty }\right]}=\lambda_{IL_{6}D}\frac{\left[D^{\max}\right]}{\left[D^{\infty }\right]}, \end{array}$$

(A55) $$\begin{array}{cc} & 2\lambda_{CA}\frac{\left[A^{\max}\right]}{\left[A^{\infty }\right]}=\lambda_{CIL_{6}}\frac{\left[IL_{6}^{\max}\right]}{\left[IL_{6}^{\infty }\right]},\;20\lambda_{EA}\frac{\left[A^{\max}\right]}{\left[A^{\infty }\right]}=\lambda_{E}, \end{array}$$

(A56) $$\begin{array}{cc} & \delta_{C}=\delta_{CT_{c}}\frac{\left[T_{c}^{\max}\right]}{\left[T_{c}^{\infty }\right]}=6\delta_{CI_{\gamma }}\frac{\left[I_{\gamma }^{\max}\right]}{\left[I_{\gamma }^{\infty }\right]}. \end{array}$$

### Appendix A.3. Parameter Values

This section includes all the parameter values used in this project. Values are separated into two groups. First, we have degradation rates included from prior research (see Table A3). Second, we have scaling-dependent parameters (see Table 1) based on the assumptions made in Appendix A.1. In order to sufficiently analyze the dynamics of the tumor based on the estimated parameters, we emphasize that possible variations in assumptions and patient to patient values should be considered. These variations have been taken into account and addressed by performing sensitivity analysis and clustering patients based on their immune profiles. In their dimensional forms, the scaling-dependent parameters would also depend on the scaling constant α, in addition to derivation assumptions and patient data. Therefore, we list the objective non-dimensional values. The dimension of these parameters is per day.

**Table A3:** **Half-lives and estimated death rate**. Degredation and death rate taken or calculated from the given references.

Parameter	Value	Reference	Parameter	Value	Reference
$\delta_{T_{N}}$	$9.49\times 10^{-4}$	[71]	$\delta_{H}$	18	[71,121]
$\delta_{T_{c}}$	0.231	[71]	$\delta_{E}$	4.16	[122]
$\delta_{T_{h}}$	0.406	[71]	$\delta_{IL_{6}}$	1.07	[71]
$\delta_{T_{r}}$	0.406	[71]	$\delta_{IL_{10}}$	4.62	[71]
$\delta_{D}$	0.277	[71]	$\delta_{IL_{12}}$	128	[123]
$\delta_{M}$	0.0198	[71]	$\delta_{I_{\gamma }}$	33.3	[71]
$\delta_{A}$	$2.8\times 10^{-3}$	[125]			

**Table A4:** **Non-dimensional parameter values for each cluster**.

Parameter	Cluster 1	Cluster 2	Cluster 3	Cluster 4	Cluster 5
${\bar{\lambda }}_{T_{h}H}$	$1.791\times 10^{-1}$	$4.048\times 10^{-2}$	$1.002\times 10^{-1}$	$2.478\times 10^{-1}$	$3.871\times 10^{-1}$
${\bar{\lambda }}_{T_{h}D}$	2.933	1.801	2.200	4.027	3.135
${\bar{\lambda }}_{T_{h}IL_{12}}$	$1.569\times 10^{-1}$	$2.286\times 10^{-2}$	$6.850\times 10^{-2}$	$2.623\times 10^{-1}$	$3.919\times 10^{-1}$
${\bar{\lambda }}_{T_{h}E}$	$1.756\times 10^{-1}$	$2.633\times 10^{-2}$	$1.063\times 10^{-1}$	$2.795\times 10^{-1}$	$4.696\times 10^{-1}$
${\bar{\lambda }}_{T_{c}E}$	6.022	3.297	4.332	8.420	7.669
${\bar{\lambda }}_{T_{c}D}$	$5.028\times 10^{-3}$	$1.128\times 10^{-2}$	$4.482\times 10^{-3}$	$6.064\times 10^{-3}$	$2.560\times 10^{-3}$
${\bar{\lambda }}_{T_{c}IL_{12}}$	$2.690\times 10^{-2}$	$1.431\times 10^{-2}$	$1.396\times 10^{-2}$	$3.950\times 10^{-2}$	$3.200\times 10^{-2}$
${\bar{\lambda }}_{T_{r}D}$	$5.783\times 10^{-2}$	$1.334\times 10^{-1}$	$6.763\times 10^{-2}$	$5.167\times 10^{-2}$	$2.721\times 10^{-2}$
${\bar{\lambda }}_{T_{r}E}$	$1.732\times 10^{-1}$	$9.755\times 10^{-2}$	$1.634\times 10^{-1}$	$1.793\times 10^{-1}$	$2.038\times 10^{-1}$
${\bar{\lambda }}_{DC}$	$3.346\times 10^{-2}$	$6.768\times 10^{-2}$	$4.237\times 10^{-2}$	$2.540\times 10^{-2}$	$2.505\times 10^{-2}$
${\bar{\lambda }}_{DH}$	$1.526\times 10^{-1}$	$1.709\times 10^{-1}$	$1.444\times 10^{-1}$	$1.474\times 10^{-1}$	$1.430\times 10^{-1}$
${\bar{\lambda }}_{DE}$	$1.497\times 10^{-1}$	$1.112\times 10^{-1}$	$1.532\times 10^{-1}$	$1.663\times 10^{-1}$	$1.734\times 10^{-1}$
${\bar{\lambda }}_{MIL_{10}}$	$2.499\times 10^{-3}$	$1.201\times 10^{-3}$	$1.978\times 10^{-3}$	$2.535\times 10^{-3}$	$3.128\times 10^{-3}$
${\bar{\lambda }}_{MI_{\gamma }}$	$1.720\times 10^{-3}$	$7.178\times 10^{-4}$	$1.244\times 10^{-3}$	$1.941\times 10^{-3}$	$2.177\times 10^{-3}$
${\bar{\lambda }}_{MIL_{12}}$	$1.991\times 10^{-3}$	$9.880\times 10^{-4}$	$1.469\times 10^{-3}$	$2.050\times 10^{-3}$	$2.484\times 10^{-3}$
${\bar{\lambda }}_{MT_{h}}$	$1.136\times 10^{-2}$	$1.575\times 10^{-2}$	$1.283\times 10^{-2}$	$1.109\times 10^{-2}$	$9.035\times 10^{-3}$
${\bar{\lambda }}_{ME}$	$2.229\times 10^{-3}$	$1.138\times 10^{-3}$	$2.279\times 10^{-3}$	$2.184\times 10^{-3}$	$2.977\times 10^{-3}$
${\bar{\lambda }}_{C}$	$5.756\times 10^{-2}$	$4.250\times 10^{-2}$	$5.669\times 10^{-2}$	$3.427\times 10^{-2}$	$3.959\times 10^{-2}$
${\bar{\lambda }}_{CIL_{6}}$	$4.345\times 10^{-4}$	$8.494\times 10^{-4}$	$3.381\times 10^{-4}$	$5.530\times 10^{-3}$	$4.595\times 10^{-3}$
${\bar{\lambda }}_{CA}$	$2.357\times 10^{-4}$	$1.243\times 10^{-3}$	$2.859\times 10^{-4}$	$1.807\times 10^{-3}$	$1.205\times 10^{-3}$
${\bar{\lambda }}_{A}$	$3.384\times 10^{-3}$	$3.394\times 10^{-3}$	$3.367\times 10^{-3}$	$3.400\times 10^{-3}$	$3.282\times 10^{-3}$
${\bar{\lambda }}_{HD}$	1.458	2.113	1.529	1.239	$7.319\times 10^{-1}$
${\bar{\lambda }}_{HN}$	4.156	2.858	2.861	3.552	4.083
${\bar{\lambda }}_{HM}$	$9.720\times 10^{-1}$	$8.862\times 10^{-1}$	1.421	$7.101\times 10^{-1}$	$7.883\times 10^{-1}$
${\bar{\lambda }}_{HT_{c}}$	3.605	4.620	4.014	7.244	6.063
${\bar{\lambda }}_{HC}$	7.809	7.523	8.173	5.255	6.334
${\bar{\lambda }}_{IL_{12}M}$	$5.253\times 10^{1}$	$4.698\times 10^{1}$	$6.480\times 10^{1}$	$3.738\times 10^{1}$	$4.730\times 10^{1}$
${\bar{\lambda }}_{IL_{12}D}$	7.880	$1.120\times 10^{1}$	6.972	6.521	4.391
${\bar{\lambda }}_{IL_{12}T_{h}}$	$4.810\times 10^{1}$	$4.533\times 10^{1}$	$3.793\times 10^{1}$	$4.596\times 10^{1}$	$3.994\times 10^{1}$
${\bar{\lambda }}_{IL_{12}T_{c}}$	$1.948\times 10^{1}$	$2.449\times 10^{1}$	$1.830\times 10^{1}$	$3.813\times 10^{1}$	$3.638\times 10^{1}$
${\bar{\lambda }}_{IL_{10}M}$	1.197	1.155	1.606	$9.978\times 10^{-1}$	1.252
${\bar{\lambda }}_{IL_{10}D}$	$1.795\times 10^{-1}$	$2.754\times 10^{-1}$	$1.728\times 10^{-1}$	$1.741\times 10^{-1}$	$1.162\times 10^{-1}$
${\bar{\lambda }}_{IL_{10}T_{r}}$	$7.419\times 10^{-1}$	$4.921\times 10^{-1}$	$5.241\times 10^{-1}$	$4.651\times 10^{-1}$	$2.271\times 10^{-1}$
${\bar{\lambda }}_{IL_{10}T_{h}}$	1.096	1.115	$9.400\times 10^{-1}$	1.227	1.057
${\bar{\lambda }}_{IL_{10}T_{c}}$	$4.440\times 10^{-1}$	$6.022\times 10^{-1}$	$4.536\times 10^{-1}$	1.018	$9.626\times 10^{-1}$
${\bar{\lambda }}_{IL_{10}C}$	$9.615\times 10^{-1}$	$9.806\times 10^{-1}$	$9.235\times 10^{-1}$	$7.384\times 10^{-1}$	1.006
${\bar{\lambda }}_{EA}$	$1.024\times 10^{-1}$	$8.334\times 10^{-2}$	$9.433\times 10^{-2}$	$1.389\times 10^{-1}$	$1.278\times 10^{-1}$
${\bar{\lambda }}_{E}$	5.935	4.761	5.600	7.870	8.708
${\bar{\lambda }}_{I_{\gamma }T_{c}}$	$2.115\times 10^{1}$	$2.278\times 10^{1}$	$2.234\times 10^{1}$	$2.611\times 10^{1}$	$2.677\times 10^{1}$
${\bar{\lambda }}_{I_{\gamma }T_{h}}$	$1.044\times 10^{1}$	8.434	9.259	6.295	5.879
${\bar{\lambda }}_{I_{\gamma }D}$	1.711	2.084	1.702	$8.931\times 10^{-1}$	$6.465\times 10^{-1}$
${\bar{\lambda }}_{IL_{6}A}$	$5.692\times 10^{-1}$	$5.110\times 10^{-1}$	$4.652\times 10^{-1}$	$5.329\times 10^{-1}$	$5.150\times 10^{-1}$
${\bar{\lambda }}_{IL_{6}M}$	$4.355\times 10^{-1}$	$4.513\times 10^{-1}$	$5.460\times 10^{-1}$	$4.573\times 10^{-1}$	$5.079\times 10^{-1}$
${\bar{\lambda }}_{IL_{6}D}$	$6.532\times 10^{-2}$	$1.076\times 10^{-1}$	$5.875\times 10^{-2}$	$7.977\times 10^{-2}$	$4.715\times 10^{-2}$
${\bar{\delta }}_{T_{h}T_{r}}$	$7.562\times 10^{-1}$	$6.086\times 10^{-1}$	$5.961\times 10^{-1}$	$6.522\times 10^{-1}$	$2.427\times 10^{-1}$
${\bar{\delta }}_{T_{h}IL_{10}}$	2.457	1.051	1.648	3.933	3.910
${\bar{\delta }}_{T_{c}IL_{10}}$	4.319	1.847	2.896	6.913	6.871
${\bar{\delta }}_{T_{c}Tr}$	1.329	1.070	1.048	1.146	$4.266\times 10^{-1}$
${\bar{\delta }}_{DC}$	$5.876\times 10^{-2}$	$7.271\times 10^{-2}$	$6.297\times 10^{-2}$	$6.209\times 10^{-2}$	$6.444\times 10^{-2}$
${\bar{\delta }}_{D_{N}}$	$2.770\times 10^{-1}$	$2.770\times 10^{-1}$	$2.770\times 10^{-1}$	$2.770\times 10^{-1}$	$2.770\times 10^{-1}$
${\bar{\delta }}_{M_{N}}$	$1.980\times 10^{-2}$	$1.980\times 10^{-2}$	$1.980\times 10^{-2}$	$1.980\times 10^{-2}$	$1.980\times 10^{-2}$
${\bar{\delta }}_{CT_{c}}$	$4.399\times 10^{-3}$	$5.276\times 10^{-3}$	$4.936\times 10^{-3}$	$8.052\times 10^{-3}$	$6.762\times 10^{-3}$
${\bar{\delta }}_{CI_{\gamma }}$	$2.741\times 10^{-3}$	$7.415\times 10^{-4}$	$1.654\times 10^{-3}$	$2.832\times 10^{-3}$	$2.982\times 10^{-3}$
${\bar{\delta }}_{C}$	$4.492\times 10^{-2}$	$3.273\times 10^{-2}$	$4.421\times 10^{-2}$	$2.606\times 10^{-2}$	$3.037\times 10^{-2}$
${\bar{\delta }}_{N}$	$2.043\times 10^{-1}$	$2.130\times 10^{-1}$	$3.031\times 10^{-1}$	$1.141\times 10^{-1}$	$1.300\times 10^{-1}$
${\bar{A}}_{T_{N}}$	9.730	5.445	7.057	$1.351\times 10^{1}$	$1.232\times 10^{1}$
${\bar{A}}_{D_{N}}$	$6.128\times 10^{-1}$	$6.267\times 10^{-1}$	$6.170\times 10^{-1}$	$6.161\times 10^{-1}$	$6.184\times 10^{-1}$
${\bar{A}}_{M}$	$3.960\times 10^{-2}$	$3.960\times 10^{-2}$	$3.960\times 10^{-2}$	$3.960\times 10^{-2}$	$3.960\cdot 10^{-2}$
${\bar{\alpha }}_{NC}$	3.924	5.497	5.966	3.089	3.240
${\bar{C}}_{0}$	9.428	7.619	8.798	8.923	8.597
${\bar{A}}_{0}$	5.795	5.713	5.937	5.666	6.813
${\bar{E}}_{0}$	3.161	6.958	3.649	2.045	1.862

### Appendix A.6. Bifurcation and Lyapunov Exponent for the Cancer ODE

To further investigate the effects of parameters on controlling the cancer development, we carried out bifurcation analysis on those involved in cancer ODE (9). This choice was made not only because these parameters were directly involved in cancer ODE, but also since they came out as the most sensitive parameters in Section 3.3. Our results show that regardless of minute differences in quantities, the qualitative behaviors are identical across all the clusters; see Figure A3.

In all clusters, for $\lambda_{C}$, $\lambda_{CIL_{6}}$, $\lambda_{CA}$, $\delta_{CT_{c}}$, $\delta_{CI_{\gamma }}$, and $\delta_{C}$, we can see the existence of a single equilibrium for a large range of parameter values (0 to 1). Increasing the values of $\lambda_{C}$, $\lambda_{CIL_{6}}$, and $\lambda_{CA}$ caused the cancer population to reach higher steady state values. However, for $\lambda_{C}$ in clusters 1–5 and for $\lambda_{CA}$ in cluster 5, we observed interesting results at the beginning of their corresponding intervals. More specifically, very small values of $\lambda_{C}$ caused the cancer population to vanish for all clusters, whereas very small values of $\lambda_{CA}$ led to very small steady state (but not zero) values for cancer only in cluster 5; see Figure A3. On the contrary, parameters $\delta_{CT_{c}}$, $\delta_{CI_{\gamma }}$ and $\delta_{C}$ only pertain to a non-vanishing cancer population in a very small parameter space. For the most part, they caused the cancer population to disappear at the steady state; see Figure A3. The negative Lyapunov exponents show that all the steady state values are Lyapunov stable.

### Appendix A.7. Positivity

In order to prove that the system with positive parameters and positive initial conditions has positive solutions, we defined the following integrating factors for each of the state variables.

$$\begin{array}{cc} \eta_{T_{h}}\left(t\right) & =\exp\left[\int_{0}^{t}\left(\delta_{T_{h}T_{r}}\left[T_{r}\right]+\delta_{T_{h}IL_{10}}\left[IL_{10}\right]+\delta_{T_{h}}\right)ds\right],\\ \eta_{T_{c}}\left(t\right) & =\exp\left[\int_{0}^{t}\left(\delta_{T_{c}T_{r}}\left[T_{r}\right]+\delta_{T_{c}IL_{10}}\left[IL_{10}\right]+\delta_{T_{c}}\right)ds\right],\\ \eta_{T_{r}}\left(t\right) & =\exp\left(\delta_{T_{r}}t\right),\\ \eta_{T_{N}}\left(t\right) & =\exp\left[\int_{0}^{t}(\lambda_{T_{h}H}\left[H\right]+\lambda_{T_{h}D}\left[D\right]+\lambda_{T_{h}IL_{12}}\left[IL_{12}\right]+\lambda_{T_{h}E}\left[E\right]\right.\\ \; & +\lambda_{T_{c}E}\left[E\right]+\lambda_{T_{c}D}\left[D\right]+\lambda_{T_{c}IL_{12}}\left[IL_{12}\right]+\lambda_{T_{r}D}\left[D\right]+\lambda_{T_{r}E}\left[E\right]+\delta_{T_{N}})ds],\\ \eta_{D_{N}}\left(t\right) & =\exp\left[\int_{0}^{t}\left(\lambda_{DC}\left[C\right]+\lambda_{DH}\left[H\right]+\lambda_{DE}\left[E\right]+\delta_{D_{N}}\right)ds\right],\\ \eta_{D}\left(t\right) & =\exp\left[\int_{0}^{t}\left(\delta_{DC}\left[C\right]+\delta_{D}\right)ds\right],\\ \eta_{M_{N}}\left(t\right) & =\exp\left[\int_{0}^{t}\left(\lambda_{MIL_{10}}\left[IL_{10}\right]+\lambda_{MI_{\gamma }}\left[I_{\gamma }\right]+\lambda_{MIL_{12}}\left[IL_{12}\right]+\lambda_{MT_{h}}\left[T_{h}\right]+\lambda_{ME}\left[E\right]+\delta_{M_{N}}\right)ds\right],\\ \eta_{M}\left(t\right) & =\exp\left(\delta_{M}t\right),\\ \eta_{C}\left(t\right) & =\exp\left[\int_{0}^{t}\left(\delta_{CT_{c}}\left[T_{c}\right]+\delta_{CI_{\gamma }}\left[I_{\gamma }\right]+\delta_{C}-\left(\lambda_{C}+\lambda_{CIL_{6}}\left[IL_{6}\right]+\lambda_{CA}\left[A\right]\right)\left(1-\frac{\left[C\right]}{C_{0}}\right)\right)ds\right],\\ \eta_{N}\left(t\right) & =\exp\left(\delta_{N}t\right),\\ \eta_{H}\left(t\right) & =\exp\left(\delta_{H}t\right),\\ \eta_{IL_{12}}\left(t\right) & =\exp\left(\delta_{IL_{12}}t\right),\\ \eta_{IL_{10}}\left(t\right) & =\exp\left(\delta_{IL_{10}}t\right),\\ \eta_{E}\left(t\right) & =\exp\left[\int_{0}^{t}\left(\delta_{E}-\lambda_{E}\left(1-\frac{\left[E\right]}{E_{0}}\right)\right)ds\right],\\ \eta_{I_{\gamma }}\left(t\right) & =\exp\left(\delta_{I_{\gamma }}t\right),\\ \eta_{IL_{6}} & =\exp\left(\delta_{IL_{6}}t\right). \end{array}$$

Considering these integration factors, we rewrote our system of ODEs as:

$$\begin{array}{cc} \frac{d}{dt}\left(\left[T_{h}\right]\eta_{T_{h}}\right) & =\left(\lambda_{T_{h}H}\left[H\right]+\lambda_{T_{h}D}\left[D\right]+\lambda_{T_{h}IL_{12}}\left[IL_{12}\right]+\lambda_{T_{h}E}\left[E\right]\right)\left[T_{N}\right]\eta_{T_{h}},\\ \frac{d}{dt}\left(\left[T_{c}\right]\eta_{T_{c}}\right) & =\left(\lambda_{T_{c}E}\left[E\right]+\lambda_{T_{c}D}\left[D\right]+\lambda_{T_{c}IL_{12}}\left[IL_{12}\right]\right)\left[T_{N}\right]\eta_{T_{c}},\\ \frac{d}{dt}\left(\left[T_{r}\right]\eta_{T_{r}}\right) & =\left(\lambda_{T_{r}D}\left[D\right]+\lambda_{T_{r}E}\left[E\right]\right)\left[T_{N}\right]\eta_{T_{r}},\\ \frac{d}{dt}\left(\left[T_{N}\right]\eta_{T_{N}}\right) & =A_{T_{N}}\eta_{T_{N}},\\ \frac{d}{dt}\left(\left[D_{N}\right]\eta_{D_{N}}\right) & =A_{D_{N}}\eta_{D_{n}},\\ \frac{d}{dt}\left(\left[D\right]\eta_{D}\right) & =\left(\lambda_{DC}\left[C\right]+\lambda_{DH}\left[H\right]+\lambda_{DE}\left[E\right]\right)\left[D_{N}\right]\eta_{D},\\ \frac{d}{dt}\left(\left[M_{N}\right]\eta_{M_{N}}\right) & =A_{M}\eta_{M},\\ \frac{d}{dt}\left(\left[M\right]\eta_{M}\right) & =\left(\lambda_{MIL_{10}}\left[IL_{10}\right]+\lambda_{MI_{\gamma }}\left[I_{\gamma }\right]+\lambda_{MIL_{12}}\left[IL_{12}\right]+\lambda_{MT_{h}}\left[T_{h}\right]+\lambda_{ME}\left[E\right]\right)\left[M_{N}\right]\eta_{M},\\ \frac{d}{dt}\left(\left[C\right]\eta_{C}\right) & =0,\\ \frac{d}{dt}\left(\left[N\right]\eta_{N}\right) & =\alpha_{NC}\left(\delta_{CI_{\gamma }}\left[I_{\gamma }\right]+\delta_{CT_{c}}\left[T_{c}\right]+\delta_{C}\right)\left[C\right]\eta_{N},\\ \frac{d}{dt}\left(\left[H\right]\eta_{H}\right) & =\left(\lambda_{HD}\left[D\right]+\lambda_{HN}\left[N\right]+\lambda_{HM}\left[M\right]+\lambda_{HT_{c}}\left[T_{c}\right]+\lambda_{HC}\left[C\right]\right)\eta_{H},\\ \frac{d}{dt}\left(\left[IL_{12}\right]\eta_{IL_{12}}\right) & =\left(\lambda_{IL_{12}M}\left[M\right]+\lambda_{IL_{12}D}\left[D\right]+\lambda_{IL_{12}T_{h}}\left[T_{h}\right]+\lambda_{IL_{12}T_{c}}\left[T_{c}\right]\right)\eta_{IL_{12}},\\ \frac{d}{dt}\left(\left[IL_{10}\right]\eta_{IL_{10}}\right) & =\left(\lambda_{IL_{10}M}\left[M\right]+\lambda_{IL_{10}D}\left[D\right]+\lambda_{IL_{10}T_{h}}\left[T_{r}\right]+\lambda_{IL_{10}T_{h}}\left[T_{h}\right]+\lambda_{IL_{10}T_{c}}\left[T_{c}\right]+\lambda_{IL_{10}C}\left[C\right]\right)\eta_{IL_{10}},\\ \frac{d}{dt}\left(\left[E\right]\eta_{E}\right) & =\lambda_{EA}\left[A\right]\eta_{E}\\ \frac{d}{dt}\left(\left[I_{\gamma }\right]\eta_{I_{\gamma }}\right) & =\left(\lambda_{I_{\gamma }T_{c}}\left[T_{c}\right]+\lambda_{I_{\gamma }T_{h}}\left[T_{h}\right]+\lambda_{I_{\gamma }D}\left[E\right]\left[D\right]\right)\eta_{I_{\gamma }},\\ \frac{d}{dt}\left(\left[IL_{6}\right]\eta_{IL_{6}}\right) & =\left(\lambda_{IL_{6}A}\left[A\right]+\lambda_{IL_{6}M}\left[M\right]+\lambda_{IL_{6}D}\left[D\right]\right)\eta_{IL_{6}}. \end{array}$$

The right-hand sides of all of the above equations are non-negative. This means that the terms $\left[X\right]\eta_{X}$ are non-decreasing for each state variable [X]. Therefore, for positive initial values, the dynamics stayed positive.
